# Expression regulation of multiple key genes to improve l-threonine in *Escherichia coli*

**DOI:** 10.1186/s12934-020-01312-5

**Published:** 2020-02-24

**Authors:** Lei Zhao, Ying Lu, Jun Yang, Yu Fang, Lifei Zhu, Zhixiang Ding, Chenhui Wang, Wenjian Ma, Xiaoqing Hu, Xiaoyuan Wang

**Affiliations:** 1 State Key Laboratory of Food Science and Technology, Jiangnan University, 1800 Lihu Avenue, Wuxi, 214122 China; 2 International Joint Laboratory on Food Safety, Jiangnan University, Wuxi, 214122 China; 3 Nanjing Customs District P. R. China, Wuxi, 214122 China; 4 Key Laboratory of Industrial Biotechnology, Ministry of Education, School of Biotechnology, Jiangnan University, Wuxi, 214122 China

**Keywords:** *Escherichia coli*, l-Threonine, Expression regulation, Metabolic flux

## Abstract

**Background:**

*Escherichia coli* is an important strain for l-threonine production. Genetic switch is a ubiquitous regulatory tool for gene expression in prokaryotic cells. To sense and regulate intracellular or extracellular chemicals, bacteria evolve a variety of transcription factors. The key enzymes required for l-threonine biosynthesis in *E. coli* are encoded by the *thr* operon. The *thr* operon could coordinate expression of these genes when l-threonine is in short supply in the cell.

**Results:**

The *thrL* leader regulatory elements were applied to regulate the expression of genes *iclR*, *arcA*, *cpxR*, *gadE*, *fadR* and *pykF*, while the threonine-activating promoters *P*_*cysH*_, *P*_*cysJ*_ and *P*_*cysD*_ were applied to regulate the expression of gene *aspC*, resulting in the increase of l-threonine production in an l-threonine producing *E. coli* strain TWF001. Firstly, different parts of the regulator *thrL* were inserted in the *iclR* regulator region in TWF001, and the best resulting strain TWF063 produced 16.34 g l-threonine from 40 g glucose after 30 h cultivation. Secondly, the gene *aspC* following different threonine-activating promoters was inserted into the chromosome of TWF063, and the best resulting strain TWF066 produced 17.56 g l-threonine from 40 g glucose after 30 h cultivation. Thirdly, the effect of expression regulation of *arcA*, *cpxR*, *gadE*, *pykF* and *fadR* was individually investigated on l-threonine production in TWF001. Finally, using TWF066 as the starting strain, the expression of genes *arcA*, *cpxR*, *gadE*, *pykF* and *fadR* was regulated individually or in combination to obtain the best strain for l-threonine production. The resulting strain TWF083, in which the expression of seven genes (*iclR*, *aspC*, *arcA*, *cpxR*, *gadE*, *pykF*, *fadR* and *aspC*) was regulated, produced 18.76 g l-threonine from 30 g glucose, 26.50 g l-threonine from 40 g glucose, or 26.93 g l-threonine from 50 g glucose after 30 h cultivation. In 48 h fed-batch fermentation, TWF083 could produce 116.62 g/L l‐threonine with a yield of 0.486 g/g glucose and productivity of 2.43 g/L/h.

**Conclusion:**

The genetic engineering through the expression regulation of key genes is a better strategy than simple deletion of these genes to improve l-threonine production in *E. coli*. This strategy has little effect on the intracellular metabolism in the early stage of the growth but could increase l-threonine biosynthesis in the late stage.

## Introduction

l-Threonine has an increasing market demand in pharmaceutical, cosmetics and food industry [1]. *Escherichia coli* is an important strain for l-threonine production [2–4]. Efforts have been made to improve the productivity of l-threonine in *E. co*li, such as increasing the carbon flux to l-threonine [5, 6], enhancing the export of l-threonine [7], weakening the competitive pathway and reducing the consumption of l-threonine [8, 9].

Genetic switch is a ubiquitous regulatory tool for gene expression in prokaryotic cells [10]. In *E. coli*, l-threonine is synthesized through a series of reactions from l-aspartate, which is converted from oxaloacetate by aspartate aminotransferase encoded by *aspC*. The key enzymes required for l-threonine biosynthesis in *E. coli* are encoded by the *thr* operon. The *thr* operon could coordinate expression of these genes when l-threonine is in short supply in the cell. This regulation is controlled by the lead sequence of the *thr* operon (*thrL*). The *thrL* contains an efficient ribosome-binding site (RBS) and can form a twenty-one-amino-acid leader peptide. From the 6th codons of this leader peptide, it starts to encode l-threonine residues, therefore, it can regulate the transcription of the *thr* operon through the availability of l-threonine, by forming different base-paired RNA structure [11]. Since *thrL* inhibits l-threonine accumulation it has been mutated in l-threonine producing *E. coli*, such as TWF001 [9]. On the other hand, some biosynthetic pathways in *E. coli* can be activated in the presence of high concentration of l-threonine, such as the sulfate metabolism branch of the cysteine biosynthetic pathway which includes the genes *cysDN*, *cysJI* and *cysH* [12].

**Fig. 1:**
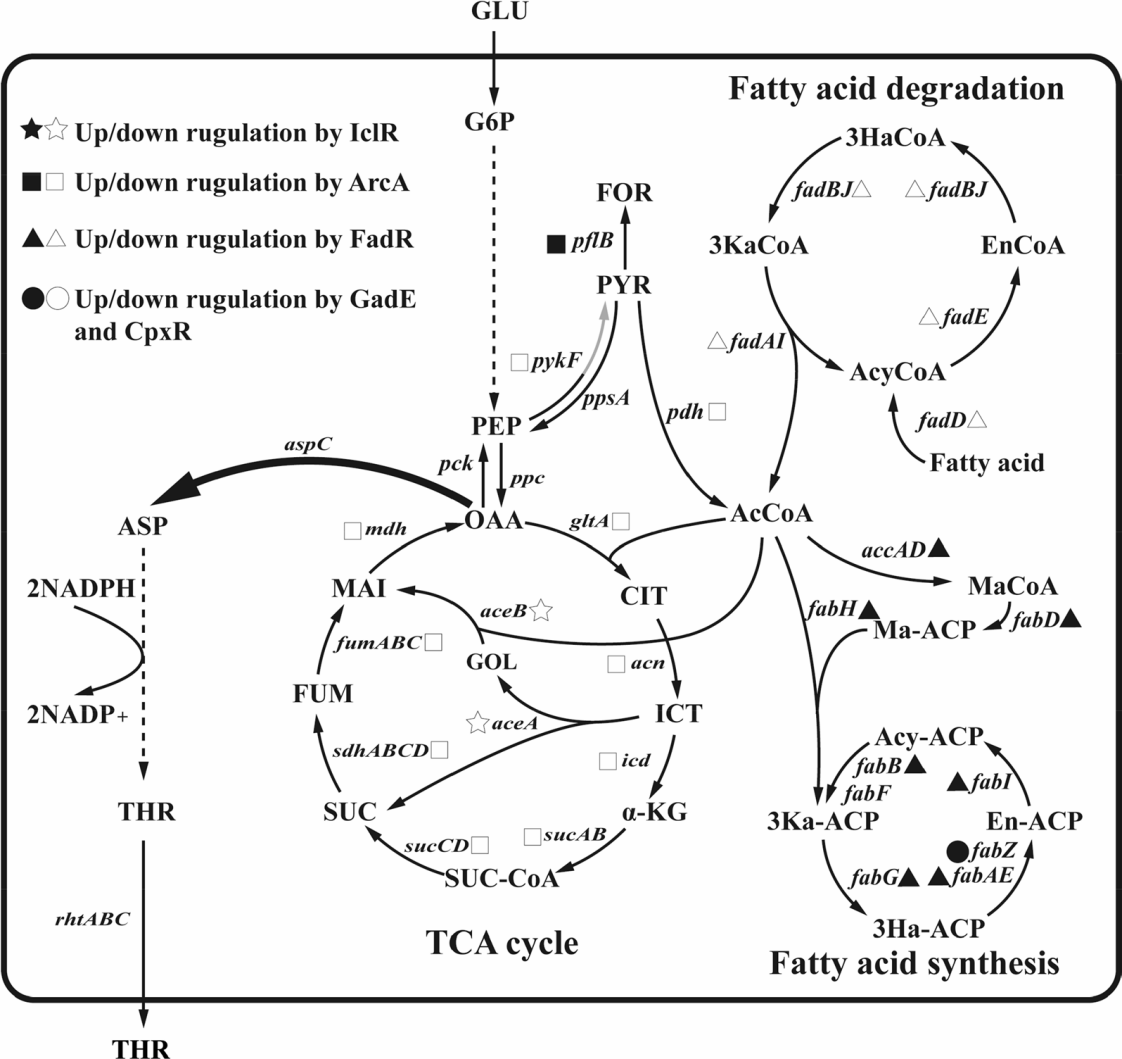
The glycolysis, TCA cycle, fatty acid metabolism, l-threonine biosynthetic pathway, and their regulation in *E. coli*. Genes encoding for the corresponding metabolic enzymes are shown in italic. The genes regulated by IclR, ArcA, FadR, GadE and CpxR are labeled by the different symbols. *GLU* glucose, *G6P* glucose-6-phosphate, *PEP* phosphoenolpyruvate, *PYR* pyruvate, *AcCoA* acetyl-coA, *CIT* citrate, *ICT* isocitrate, *α-KG* α-ketoglutarate, *SUC-CoA* succinyl-CoA, *SUC* succinate, *FUM* fumarate, *MAL* malate, *OAA* oxaloacetate, *ASP* aspartate, *THR* threonine, *FOR* formate, *Acy-ACP* Acyl-ACP, *En-ACP* Enoyl-ACP, *3Ha-ACP* 3-Hydroxyacyl-ACP, *3Ka-ACP* 3-Ketoacyl-ACP, *MaCoA* Malonyl-CoA, *3HaCoA* 3-Hydroxyacyl-CoA, *EnCoA* Enoyl-CoA, *AcyCoA* Acyl-CoA, *3KaCoA* 3-Ketoacyl-CoA

To sense and regulate intracellular or extracellular chemicals, bacteria evolve a variety of transcription factors. Based on this principle, various biosensors for amino acids and their precursors, including l-methionine, l-leucine, l-isoleucine, l-valine [13, 14], l-lysine, l-arginine, l-serine, *O*-acetyl-l-serine [15], *O*-acetyl homoserine [16], and oxygen [17] have been constructed, and some of these sensors have been successfully applied to the high-throughput screening [12, 18, 19] and systems metabolic engineering [20]. l-Threonine production in *E. coli* can be enhanced by regulating IclR [8, 9], a transcription factor. IclR is a repressor for the *aceBAK* operon (Fig. 1), which encodes the three enzymes in the glyoxylate bypass [21]. The deletion of *iclR* in *E. coli* can improve production of phloroglucinol and 3-hydroxypropionate [22], produce succinate from acetate [23], and produce fumaric acid [24, 25], ectoine [26] and fatty acids [27]. The metabolism regulator protein FadR represses the transcription of the *fadBA* operon, *fadE*, *fadD*, *fadH*, *fadIJ* operon, *fadL* and *fadM*, which involved in the fatty acid degradation [28]. FadR can also activate the express of gene *iclR* and the genes *accA*, *accD*, *accBC* operon, *fabA*, *fabB* and *fabHDG* operon involved in the fatty acid biosynthesis (Fig. 1). Therefore, the absence of FadR can weaken the lipid biosynthesis and enhance the fatty acid degradation in *E. coli* [29–31]. The *iclR* deletion mutant strain TWF006 further modified by enhancing fatty acid degradation and the glyoxylate shunt can increase l-threonine production [6]. CpxR and GadE can also activate the genes involved in the dissociated or type II fatty acid synthase systems, such as *fabA* and *fabZ* [32, 33]. Under the anaerobic conditions, ArcA represses the genes involved in TCA cycle and glyoxylate shunt [34], such as *gltA* [35], *acnAB* [36], *icd* [37], *sucABCD* [38], *lpd* [39], *sdhCDAB* [40], *fumA*, *fumC* [41], *fumB* [42], *mdh* [43], *maeA* and *aceAB* [44], and the key genes *aceEF* and *lpdA* [45] which encoding the enzymes reaction from pyruvate to acetyl-CoA. The l-threonine production in *E. coli* strain TWF001 was increased when *arcA* and *iclR* were deleted [46]. Expressing two pyruvate kinase isoenzymes, PYKI and PYKII, encoded by *pykF* and pykA, respectively, is important for cellular metabolism and glycolysis in *E. coli*. In *pykF* deletion mutant, the flux of Embden-Meyerhof-Parnas pathway was reduced from 65 to 20% while the flux of pentose phosphate pathway was increased from 34 to 79% [47]. Acetate, the main by-product of *E. coli* at high cell density, can inhibit the cell growth and l-threonine production. Since acetate formation is correlated with the metabolic flux overflow, *pykF* deletion can lead to less acetate and more l-threonine production in an l-threonine-producing *E. coli* THRD [48]. In this study, l-threonine was used to regulate the expression of genes *iclR*, *arcA*, *cpxR*, *gadE*, *fadR*, *pykF*, and *aspC* through *thrL* and the promoters of *cysD*, *cysH* and *cysJ*, leading to the increased l-threonine production in *E. coli* TWF001 (Fig. 1). The expression of these seven genes in *E. coli* are controlled by the concentration of l-threonine. The final strain TWF083 constructed in this study could produce 26.50 g/L l-threonine after 21 h flask fermentation, which was 49.4% increase compared to the control strain TWF001.

## Materials and method

### Chemicals and reagents

Plasmids and genomic DNA were extracted using the Plasmids mini kit and DNA purification kit (Tiangen, Beijing, China), respectively. PrimerSTAR HS DNA polymerase (Takara, Dalian, China) was used for PCR. Restriction endonucleases and T4 DNA ligase were purchased from Thermo Scientific (Waltham, USA). Total RNA was extracted using RNA extraction kit (Bio Flux, Beijing, China). The RNA was reversely transcribed into cDNA using the Revert Aid™ First Strand cDNA synthesis kit (Fermentas, Shanghai, China). RT-PCR was performed using the Real Master Mix kit (Tiangen, Beijing, China). The intracellular concentration of NADPH/NADP^+^ was determined by using NADPH/NADP^+^ Kit (Beyotime Biotechnology, Shanghai, China).

### Bacterial growth condition

The bacterial strains and plasmids used in this study are listed in Table 1. The primers used in this study are listed in Table 2. The promoters used in this study are listed in Table 3. The ribosome binding sites (RBSs) used in this study are listed in Table 4. The Luria–Bertani (LB) medium (5 g/L yeast extract, 10 g/L tryptone, and 10 g/L NaCl) was used to culture the *E. coli* cells at 37 °C or 30 °C with 200 rpm shaking. To maintain the plasmids in the engineered strains or to provide the selective pressure, kanamycin (50 mg/L), spectinomycin (50 mg/L), isopropyl-d-thiogalactopyranoside (IPTG; 0.5 mM) or arabinose (10 mM) was supplemented in the medium.

**Table 1:** Bacterial trains and plasmids used in this study

Strains or plasmids	Description	Sources
Strains		
JM109	Wild type *E. coli*	NEB
MG1655	Wild type *E. coli* K-12; F^−^λ^−^ rph-1	CGSC 6300
TWF001	l-Threonine-producing *E. coli* strain	[9]
TWF003	TWF001Δ*iclR*	[9]
TWF010	TWF001Δ*arcA*	[46]
TWF051	Derived from TWF001 by replacing *RBS*_*arcA*_ (the *RBS* sequence of *arcA*) with *thrR* (*RBS*_*thrL*_-*thrL*-*RBS*_*thrA*_, the *RBS* sequence of *thrL* connected with the DNA sequence of the *thr* operon leader regions, *thrL*, and followed by the *RBS* sequence of *thrA*)	This study
TWF052	Derived from TWF001 by replacing *RBS*_*cpxR*_ with *thrR*	This study
TWF053	Derived from TWF001 by replacing *RBS*_*gadE*_ with *thrR*	This study
TWF054	Derived from TWF001 by replacing *RBS*_*pykF*_ with *thrR*	This study
TWF055	Derived from TWF001 by replacing *RBS*_*fadR*_ with *thrR*	This study
TWF056	Derived from TWF001 by inserting the DNA sequence *thrR*_*1*_ (the DNA sequence of the *thr* operon leader regions, *thrL*, followed by the *RBS* sequence of *thrA*) between *RBS*_*iclR*_ (the *RBS* sequence of *iclR*) and gene *iclR*	This study
TWF057	Derived from TWF001 by inserting the DNA sequence *thrR*_*2*_ (the *RBS* sequence of *thrL*, followed by *thrL*) between *P*_*iclR*_ (the *Promoter* sequence of *iclR*) and *RBS*_*iclR*_	This study
TWF058	Derived from TWF001 by replacing *RBS*_*iclR*_ with the DNA sequence *thrR*	This study
TWF059	Derived from TWF001 by replacing *P*_*iclR*_ and *RBS*_*iclR*_ with the DNA sequence *P*_*thrl*_-*thrR*	This study
TWF060	Derived from TWF058 by replacing *RBS*_*iclR*_ with *thrR*_*s1*_ (*RBS*_*thrL*_-*thrL*-*RBS*_*s1*_)	This study
TWF061	Derived from TWF058 by replacing *RBS*_*iclR*_ with *thrR*_*s2*_ (*RBS*_*s2*_-*thrL*-*RBS*_*thrA*_)	This study
TWF062	Derived from TWF058 by replacing *RBS*_*iclR*_ with *thrR*_*s3*_ (*RBS*_*s3*_-*thrL*-*RBS*_*thrA*_)	This study
TWF063	Derived from TWF058 by replacing *RBS*_*iclR*_ with *thrR*_*s4*_ (*RBS*_*s4*_-*thrL*-*RBS*_*thrA*_)	This study
TWF064	Derived from TWF058 by replacing *RBS*_*iclR*_ with *thrR*_*s5*_ (*RBS*_*s5*_-*thrL*-*RBS*_*thrA*_)	This study
TWF065	Derived from TWF058 by replacing *RBS*_*iclR*_ with *thrR*_*s6*_ (*RBS*_*s6*_-*thrL*-*RBS*_*thrA*_)	This study
TWF066	Derived from TWF063 by inserting the DNA sequence *P*_*cysH*_-*RBS*_*s7*_-*aspC* (the *Promoter* sequence of *cysH* followed by a synthetic *RBS*_*s7*_ and the gene *aspC*) in the *lacI* locus	This study
TWF067	Derived from TWF063 by inserting a DNA sequence *P*_*cysJ*_-*RBS*_*s8*_-*aspC* in the *lacI* locus	This study
TWF068	Derived from TWF063 by inserting a DNA sequence *P*_*cysD*_-*RBS*_*s9*_-*aspC* in the *lacI* locus	This study
TWF069	Derived from TWF066 by replacing *RBS*_*arcA*_ with *thrR*	This study
TWF070	Derived from TWF066 by replacing *RBS*_*cpxR*_ with *thrR*	This study
TWF071	Derived from TWF066 by replacing *RBS*_*gadE*_ with *thrR*	This study
TWF072	Derived from TWF066 by replacing *RBS*_*pykF*_ with *thrR*	This study
TWF073	Derived from TWF066 by replacing *RBS*_*fadR*_ with *thrR*	This study
TWF074	Derived from TWF070 by replacing *RBS*_*arcA*_ with *thrR*	This study
TWF075	Derived from TWF070 by replacing *RBS*_*gadE*_ with *thrR*	This study
TWF076	Derived from TWF070 by replacing *RBS*_*pykF*_ with *thrR*	This study
TWF077	Derived from TWF070 by replacing *RBS*_*fadR*_ with *thrR*	This study
TWF078	Derived from TWF077 by replacing *RBS*_*arcA*_ with *thrR*	This study
TWF079	Derived from TWF077 by replacing *RBS*_*gadE*_ with *thrR*	This study
TWF080	Derived from TWF077 by replacing *RBS*_*pykF*_ with *thrR*	This study
TWF081	Derived from TWF078 by replacing *RBS*_*gadE*_ with *thrR*	This study
TWF082	Derived from TWF078 by replacing *RBS*_*pykF*_ with *thrR*	This study
TWF083	Derived from TWF081 by replacing *RBS*_*pykF*_ with *thrR*	This study
Plasmids		
pCas9	*repA*1*0*1*(Ts) kan P*_*cas*_-*cas9 P*_*araB*_-*Red lacIq P*_*trc*_-sgRNA-*pMB*1	[49]
pTargetF	*pMB*1 *aadA* sgRNA	[49]
pTargetF-*arcA*	*pMB*1 *aadA sgRNA*-*arcA*	This study
pTargetF-*cpxR*	*pMB*1 *aadA sgRNA*-	This study
pTargetF-*gadE*	*pMB*1 *aadA sgRNA*-*gadE*	This study
pTargetF-*pykF*	*pMB*1 *aadA sgRNA*-*pykF*	This study
pTargetF-*fadR*	*pMB*1 *aadA sgRNA*-*fadR*	This study
pTargetF-*iclR1*	*pMB*1 *aadA sgRNA*-*iclR1*	This study
pTargetF-*iclR2*	*pMB*1 *aadA sgRNA*-*iclR2*	This study
pTargetF-*lacI*	*pMB*1 *aadA sgRNA*-*lacI*	This study

**Table 2:** The primers used in this study

Names	Sequence (5′-3′)	Purpose
P*arcA*-U-FR1	TGTTAATTTGCAGCATGCATCAGGCAGGTCAGGGACTTTTGATAGCGCACAGACAGATAAAAATTACA	The *thrR*-*arcA* fragments with the homologous arm of *arcA*
P*arcA*-D-RF1	TGTTACCAACTCGTCTTCAACGATAAGAATGTGCGGGGTCTGCATGGTTGTTACCTCGTTACCTTTGG	
P*arcA*-U-FR11	AACGCAATTACGTACTTTAGTCATGTTACGCCGATCATGTTAATTTGCAGCATGCATCA	
P*arcA*-D-RF11	GCCTTCCGCTTCGAAAATACTTTTCAACGTGTTGCGTGTTACCAACTCGTCTTCAACGA	
Pf-P*arcA*-F	GTCCTAGGTATAATACTAGT TTCGATTTAGTTGGCAATTTGTTTTAGAGCTAGAAATAG	pTargetF-*arcA*
pf-P*arcA*-R	CTATTTCTAGCTCTAAAACAAATTGCCAACTAAATCGAAACTAGTATTATACCTAGGAC	
Pf-P*arcA*-V-F	TTCGATTTAGTTGGCAATTT	
P*arcA*-V-R	CGGGTTGAACGGTTTGGTG	Identify and sequence TWF051, TWF069, TWF074 and TWF078
P*arcA*-S-F	CGGGTTGAACGGTTTGGTG	
P*arcA*-S-R	CGGGTTGAACGGTTTGGTG	
P*cpxR*-U-FR	AAGTCATGGATTAGCGACGTCTGATGACGTAATTTCTGCCTATAGCGCACAGACAGATAAAAATTACA	The *thrR*-*cpxR* fragments with the homologous arm of *cpxR*
P*cpxR*-D-RF	GGAAGTCAGCTCTCGGTCATCATCAACTAACAGGATTTTATTCATGGTTGTTACCTCGTTACCTTTGG	
P*cpxR*-U-FR11	GCTGCAAACATGCGTCAGGGGGTGTAAAACAACGTAAAGTCATGGATTAGCGACGTCTG	
P*cpxR*-D-RF11	CGTTGAAGCCTTCCATCTCGAGCAGCTCCTTTAATAGGGAAGTCAGCTCTCGGTCATCA	
Pf-P*cpxR*-F	GTCCTAGGTATAATACTAGTTGACGTAATTTCTGCCTCGGGTTTTAGAGCTAGAAATAG	pTargetF-*cpxR*
pf-P*cpxR*-R	CTATTTCTAGCTCTAAAACCCGAGGCAGAAATTACGTCAACTAGTATTATACCTAGGAC	
Pf-P*cpxR*-V-F	TGACGTAATTTCTGCCTCGG	
P*cpxR*-V-R	GCCGCCACCACATTAA	Identify and sequence TWF052 and TWF070
P*cpxR*-S-F	AGCAGCGTGGCTTAATGAACT	
P*cpxR*-S-R	CATAATGACAGGCGTCTGGTGT	
P*gadE*-U-FR	ACAAGGATGTAAATAATGAAAAGGATGACATATTCGAAACGATAGCGCACAGACAGATAAAAATTACA	The *thrR*-*gadE* fragments with the homologous arm of *gadE*
P*gadE*-D-RF	AAAGCCCTGTAAAAGAAAAGAATCTTTCGTCATGAGAAAAATCATGGTTGTTACCTCGTTACCTTTGG	
P*gadE*-U-FR11	ATAGGCGTTTACTATATTGAACAACGATTCGGACAAGGATGTAAATAATGAAAAGGATG	
P*gadE*-D-RF11	TTTTATCATTTCGTGATTATCTTTCAACTGCCAAAAGCCCTGTAAAAGAAAAGAATCTT	
Pf-P*gadE*-F	GTCCTAGGTATAATACTAGTATTCGAAACGATAACGGCTAGTTTTAGAGCTAGAAATAG	pTargetF-*gadE*
pf-P*gadE*-R	CTATTTCTAGCTCTAAAACTAGCCGTTATCGTTTCGAATACTAGTATTATACCTAGGAC	
Pf-P*gadE*-V-F	ATTCGAAACGATAACGGCTA	
P*gadE*-V-R	GTTCCTGCCAGCATTCG	Identify and sequence TWF053, TWF071, TWF075, TWF079 and TWF081
P*gadE*-S-F	TGATAACTTATTCTTGGGCAGTA	
P*gadE*-S-R	TTCATCAAGGATATGATTGTG	
P*pykF*-U-FR	ACCGGATTCGCTTTCCGGCAGTGCGCCCAGAAAGCAAGTTTATAGCGCACAGACAGATAAAAATTACA	The *thrR*-*pykF* fragments with the homologous arm of *pykF*
P*pykF*-D-RF	TTCGGTTTTCGGTCCGATGGTGCAAACAATTTTGGTCTTTTTCATGGTTGTTACCTCGTTACCTTTGG	
P*pykF*-U-FR11	TGTCACCTATCCTTAGAGCGAGGCACCACCACTTTCGTAATACCGGATTCGCTTTCCGG	
P*pykF*-D-RF11	TCATGCCAGCGTCCAGCATTTTAGCTAACATCTCTTCAGATTCGGTTTTCGGTCCGATG	
Pf-P*pykF*-F	GTCCTAGGTATAATACTAGTTCTTAGTCTTTAAGTTGAGAGTTTTAGAGCTAGAAATAG	pTargetF-*pykF*
pf-P*pykF*-R	CTATTTCTAGCTCTAAAACTCTCAACTTAAAGACTAAGAACTAGTATTATACCTAGGAC	
Pf-P*pykF*-V-F	TCTTAGTCTTTAAGTTGAGA	
P*pykF*-V-R	ACTTCCATACCGATCAGACCAT	Identify and sequence TWF054, TWF072, TWF076, TWF080, TWF082 and TWF083
P*pykF*-S-F	TGACAACTTCGGCACCAGA	
P*pykF*-S-R	GTCGTTACCGCCTTCCAGT	
P*fadR*-U-FR	GCGTAGTTAGCCCTCTGGTATGATGAGTCCAACTTTGTTTATAGCGCACAGACAGATAAAAATTACA	The *thrR*-*fadR* fragments with the homologous arm of *fadR*
P*fadR*-D-RF	GAAACCCGCCGGGCTTTGCGCCTTAATGACCATGGTTGTTACCTCGTTACCTTTGG	
P*fadR*-U-FR11	GCCTTGATCCCTTTTTCTTCTTTTTGTCTGCTATCAGCGTAGTTAGCCCTCTGGTATGA	
P*fadR*-D-RF11	AAGCGGTTATTCCAGATACTTTCAATAATGTACTCTTCCGCGAAACCCGCCGGGCTTTG	
Pf-P*fadR*-F	GTCCTAGGTATAATACTAGTAACTTTGTTTTGCTGTGTTAGTTTTAGAGCTAGAAATAG	pTargetF-*fadR*
pf-P*fadR*-R	CTATTTCTAGCTCTAAAACTAACACAGCAAAACAAAGTTACTAGTATTATACCTAGGAC	
Pf-P*fadR*-V-F	AACTTTGTTTTGCTGTGTTA	
P*fadR*-V-R	GCCAAATCTCGCCACTC	Identify and sequence TWF055, TWF073 and TWF077
P*fadR*-S-F	GAGATCTCCATGATGGTTTCCCTTA	
P*fadR*-S-R	GAGATCTCCATGATGGTTTCCCTTA	
P*iclR*-U-FR1	TGAAAATGATTTCCACGATACAGAAAAAAGAGACTGTCATGAAACGCATTAGCACCACC	The *thrR*_*1*_ fragments with the homologous arm of *iclR*
P*iclR*-D-RF1	TTTTCTGCCGCGTTTCGCGGGAATGGGTGCGACCATGGTTGTTACCTCGTTACCTTTGG	
P*iclR*-U-FR11	GTTCAGTAACTATTGCATTAGCTAACAATAAAAATGAAAATGATTTCCACGATACAGAA	
P*iclR*-D-RF11	GACTGAACCTGTCCAGTCGCTGGTGCGGTGGCAACGGCGGGTTTTCTGCCGCGTTTCGC	
Pf-P*iclR*-F	GTCCTAGGTATAATACTAGTACAGAAAAAAGAGACTGTCAGTTTTAGAGCTAGAAATAG	pTargetF-*iclR1*
pf-P*iclR*-R	CTATTTCTAGCTCTAAAACTGACAGTCTCTTTTTTCTGTACTAGTATTATACCTAGGAC	
Pf-P*iclR*-V-F	ACAGAAAAAAGAGACTGTCA	
pf-V-R	TTGTCAGCAAGATAGCCAGA	
ThrL-V-F	ATGAAACGCATTAGCACC	Identify and sequence TWF056, TWF057, TWF058, TWF059, TWF060, TWF061, TWF062, TWF063, TWF064 and TWF065
P*iclR*-V-R	CACCTGTTCTTCGCTCAGTTGG	
P*iclR*-S-F	GTAGGCGTTGTGGATAGCGG	
P*iclR*-S-R	TTGCCCAATGTCCCAGTTCG	
P*iclR*-U-FR2	ATTAGCTAACAATAAAAATGAAAATGATTTCCATAGCGCACAGACAGATAAAAATTACA	The *thrR*_*2*_ fragments with the homologous arm of *iclR*
P*iclR*-D-RF2	ATGGGTGCGACCATGACAGTCTCTTTTTTCTGTATCGTAAAAAAAAAGCCCGCACTGTC	
P*iclR*-U-FR22	ACTCATCGGATCAGTTCAGTAACTATTGCATTAGCTAACAATAAAAATGAAAATGATTT	
P*iclR*-D-RF22	GCGGTGGCAACGGCGGGTTTTCTGCCGCGTTTCGCGGGAATGGGTGCGACCATGACAGT	
Pf-P*iclR*-F2	GTCCTAGGTATAATACTAGTAGTCTCTTTTTTCTGTATCGGTTTTAGAGCTAGAAATAG	pTargetF-*iclR2*
pf-P*iclR*-R2	CTATTTCTAGCTCTAAAACCGATACAGAAAAAAGAGACTACTAGTATTATACCTAGGAC	
Pf-P*iclR*-V-F2	AGTCTCTTTTTTCTGTATCG	
P*iclR*-U-FR3	CACCACGCAACATGAGATTTGTTCAACATTAAAAAACGCCTTAGTAAGTATTTTTCAGC	*P*_*thrL*_- *thrR* fragments with the homologous arm of *iclR*
P*iclR*-U-FR33	ACCATACTGGCATAAACGCATCTGTGGTAAAAGCGACCACCACGCAACATGAGATTTGT	
P*iclR*-D-RF4	TTTCGCGGGAATGGGTGCGACCATTGAGATACCTCTTCTTATTTTTCTGAGCAAAAAAAAAGCCCGCACTGTC	The *thrR*_*s1*_ fragments with the homologous arm of *iclR*
P*iclR*-D-RF44	CCTGTCCAGTCGCTGGTGCGGTGGCAACGGCGGGTTTTCTGCCGCGTTTCGCGGGAATGGGTGC	
P*iclR*-U-FR5	ATTAGCTAACAATAAAAATGAAAATGATTTCCACCAAAACCATAAGGAGGATTTACATGAAACGCATTAGCACCACC	The *thrR*_*s2*_ fragments with the homologous arm of *iclR*
P*iclR*-U-FR55	ACATGAGATTTGTTCAACATTAACTCATCGGATCAGTTCAGTAACTATTGCATTAGCTAACAATAAAAATGAAAATGATTT	
P*iclR*-U-FR6	ATTAGCTAACAATAAAAATGAAAATGATTTCCGTAGCTGAGACTCTAAGGGAGGCGTCACATGAAACGCATTAGCACCACC	The *thrR*_*s3*_ fragments with the homologous arm of *iclR*
P*iclR*-U-FR7	ATTAGCTAACAATAAAAATGAAAATGATTTCCGCATACATTCCACGACCATAGGAACCAACCCATGAAACGCATTAGCACCACC	The *thrR*_*s4*_ fragments with the homologous arm of *iclR*
P*iclR*-U-FR8	ATTAGCTAACAATAAAAATGAAAATGATTTCCAAGCCCATTTTACCATCGAAGAGGGACAGAATGAAACGCATTAGCACCACC	The *thrR*_*s5*_ fragments with the homologous arm of *iclR*
P*iclR*-U-FR9	ATTAGCTAACAATAAAAATGAAAATGATTTCCTCCTTTTAGGGCTATCGCGGAGGAGTAATGAAACGCATTAGCACCACC	The *thrR*_*s6*_ fragments with the homologous arm of *iclR*
*cysH*-*aspC*-F2	TAGCCACCTAGGAGGACTCCATGTTTGAGAACATTACCGCCG	The *RBS*_*s7*_-*aspC* fragment
*cysH*-*aspC*-R2	CTTCCACTTTTTCCCGCGTTTTCGCAGAAACGTGGCTGGCCTGGTTCACCACGCGGGAAACGGTCTGATAGGCACATAAAAAAGCCCGC	
*cysH*-*aspC*-F1	ACCTTTCGCGGTATGGCATGATAGCGCCCGGAAGAGAGTCAATTCAGGGTGGTGAATGTGAAACCAGTACGGAAATCCTGGCGTCGC	The promoter of *cysH*
*cysH*-*aspC*-R1	GGAGTCCTCCTAGGTGGCTATGCCTTGCCTGATGCGAC	
cys-*aspC*-F	TTCTGGTGGCCGGAAGGCGAAGCGGCATGCATTTACGTTGACACCATCGAATGGCGCAAAACCTTTCGCGGTATGGCAT	The *P*_*cysH*_-*RBS*_*s7*_-*aspC* fragment with the homologous arm of *lacI*
cys-*aspC*-R	TGTTTGCCCGCCAGTTGTTGTGCCACGCGGTTGGGAATGTAATTCAGCTCCGCCATCGCCGCTTCCACTTTTTCCCGCG	
Pf-*lacI*-F	GTCCTAGGTATAATACTAGTTACGATGTCGCAGAGTATGCGTTTTAGAGCTAGAAATAG	pTargetF-*lacI*
pf-*lacI*-R	CTATTTCTAGCTCTAAAACGCATACTCTGCGACATCGTAACTAGTATTATACCTAGGAC	
Pf-*lacI*-V-F	TACGATGTCGCAGAGTATGC	
*lacI*-aspC-V-F	CCTACGCTGGAACAATGGCAAACACTGGCACA	Identify and sequence TWF066, TWF067 and TWF068
*lacI*-aspC-V-R	GCTTCCACAGCAATGGCATCCTGGTCATCC	
*lacI*-S-F	AACAGATCGAAGAAGGGGTTGA	
*lacI*-S-R	GACGGCGCGTGCAGGGCC	
*cysJ*-*aspC*-F2	CGCATAACAATTCGTATCATAAGGAGACCATATGTTTGAGAACATTACCGCCG	The *P*_*cysJ*_-*RBS*_*s8*_-*aspC* fragment with the homologous arm of *lacI*
*cysJ*-*aspC*-F1	ACCTTTCGCGGTATGGCATGATAGCGCCCGGAAGAGAGTCAATTCAGGGTGGTGAATGTGAAACCAGTAGTTGCGCAAAATCGCTGATT	
*cysJ*-*aspC*-R1	ATGGTCTCCTTATGATACGAATTGTTATGCGGTAAGCAAAGCTGTTTCTGCGC	
*cysD*-*aspC*-F2	AAGGCCAAAGACACTAAGAATTATTTATATGTTTGAGAACATTACCGCCG	The *P*_*cysD*_-*RBS*_*s9*_-*aspC* fragment with the homologous arm of *lacI*
*cysD*-*aspC*-F1	CCTTTCGCGGTATGGCATGATAGCGCCCGGAAGAGAGTCAATTCAGGGTGGTGAATGTGAAACCAGTACGGTGCCTTAAGCACTTTTTG	
*cysD*-*aspC*-R1	ATAAATAATTCTTAGTGTCTTTGGCCTTATGTTTCGACTATAGGGAGCGTAAG	
RT-*16SrDNA*-F	TTTAATTCGATGCAACGCGAAGAACC	Transcriptional level
RT-*16SrDNA*-R	CGGACCGCTGGCAACAAAGGATAAG	
RT-*iclR*-F	CAGGGTTTCGTGCGTCAGGTTGG	
RT-*iclR*-R	CGATAATAATCGCTTCGTGATCGCTTTG	
RT-*aspC*-F	GAGAACATTACCGCCGCTCCTGC	
RT-*aspC*-R	CCCGTCTCATCTTTATAGACACCAATCCC	
RT-*fadR*-F	GGAAGTGCTGGCTACCGCTAATGAAG	
RT-*fadR*-R	CATCCCGTTAAGAATCAGACCGTAAATCG	
RT-*cpxR*-F	ACAGCATCTGGGTCAGGTGGTTTCC	
RT-*cpxR*-R	CGGCAGTTTACGACGCAGGTTGG	
RT-*gadE*-F	AGGCAATAAACCCTTCAAG	
RT-*gadE*-R	TCGGCATCTAATTTCTCCAG	
RT-*pykF*-F	GCGTTTCCATTGCTCTGCC	
RT-*pykF*-R	GCTTTCAGGTGCTCACGGATTTC	
RT-*arcA*-F	AGTCCCGTGCTGAACTGCTGAAG	
RT-*arcA*-R	ACCGTGAATGGTGGCGATG

**Table 3:** The promoters used in this study

Promoter	Sequence (5′-3′)
*thrL*	GCCGTGAGTAAATTAAAATTTTATTGACTTAGGTCACTAAATACTTTAACCAATATAGGC
*iclR*	CTCATCGGATCAGTTCAGTAACTATTGCATTAGCTAACAATAAAAATGAAAATGATTTCC
*cysH*	CGGAAATCCTGGCGTCGCTTGATGAACTGATAGGGCGCTGGGCGAAAGAGCGCGAAGCGGGTGAAGGCTTCGGCGACTTTACGGTGCGTGCGGGCATCATTCGCCCGGTGCTCGATCCGGCGCGTGATTTGTGGGATTAACCATCAGCCCGGTCTTGTAGGCCTGATAAGAACGCGTGAGCGTCGCATCAGGCAAGGCA
*cysJ*	GTTGCGCAAAATCGCTGATTTATCTTAATGATTGGCTAAATTCATTTGTTTTTCATTAGGTTGGTTAATCTATTTTGTTGTTAAAGACTATTGCTAAAACAGGTTAGTCGATTTGGTTATTAGTTATCGCTATCCCGTCTTTAATCCACACCGTTTGCCCCGTTAACCTTACCTTCTCTTCTGTTTTATGGGCGCTGACAGGGCGCAGAAACAGCTTTGCTTAC
*cysD*	CGGTGCCTTAAGCACTTTTTGATATTAGCTTTGCCAAATCGTTATTCCGTTAAGGAACTACTCATTCTAATTGGTAATTTCATTCGTTCTCTTACGCTCCCTATAGTCGAAACAT
*arcA*	ATCATGTTAATTTGCAGCATGCATCAGGCAGGTCAGGGACTTTTG
*cpxR*	GGGGGTGTAAAACAACGTAAAGTCATGGATTAGCGACGTCTGATGACGTAATTTCTGCCT
*gadE*	ATATTGAACAACGATTCGGACAAGGATGTAAATAATGAAAAGGATGACATATTCGAAACG
*pykF*	ATATTTTTTGAAACGCTGTTTTTGTTTTCCTTTTGGATTAATTTCAGCGTATAATGCGCGCCAATTGACTCTTGAATGGTTTCAGCACTTTGGACTGTAGAACTCAACGACTCAAAAACAGGCACTCACGTTGGGCTGAGACACAAGCACACATTCCTCTGCACGCTTTTTCGATGTCACCTATCCTTAGAGCGAGGCACCACCACTTTCGTAATACCGGATTCGCTTTCCGGCAGTGCGCCCAGAAAGCAAGTTT
*fadR*	ATCCCTTTTTCTTCTTTTTGTCTGCTATCAGCGTAGTTAGCCCTCTGGTATGATGAGTCCAACTTTGTTT

**Table 4:** The ribosome binding sites used in this study

Names	Sequence (5′-3′)	RBS Calculator’s proportional scale (au) [64]
*RBS*_*thrL*_	ATAGCGCACAGACAGATAAAAATTACAGAGTACACAACATCC	–
*RBS*_*thrA*_	CGACCAAAGGTAACGAGGTAACAACC	–
*RBS*_*iclR*_	ACGATACAGAAAAAAGAGACTGTC	–
*RBS*_*s1*_	GCTCAGAAAAATAAGAAGAGGTATCTCA	3800
*RBS*_*s2*_	ACCAAAACCATAAGGAGGATTTAC	380,000
*RBS*_*s3*_	GTAGCTGAGACTCTAAGGGAGGCGTCAC	45000
*RBS*_*s4*_	GCATACATTCCACGACCATAGGAACCAACCC	15,000
*RBS*_*s5*_	AAGCCCATTTTACCATCGAAGAGGGACAGA	5000
*RBS*_*s6*_	TCCTTTTAGGGCTATCGCGGAGGAGTA	1000
*RBS*_*s7*_	TAGCCACCTAGGAGGACTCC	2000
*RBS*_*s8*_	CGCATAACAATTCGTATCATAAGGAGACCAT	2000
*RBS*_*s9*_	AAGGCCAAAGACACTAAGAATTATTTAT	2000
*RBS*_*arcA*_	TACTTCCTGTTTCGATTTAGTTGGCAATTTAGGTAGCAAAC	–
*RBS*_*cpxR*_	CGGAGGTATTTAAACA	–
*RBS*_*gadE*_	ATAACGGCTAAGGAGCAAGTT	–
*RBS*_*pykF*_	CTCCCATCCTTCTCAACTTAAAGACTAAGACTGTC	–
*RBS*_*fadR*_	TGCTGTGTTATGGAAATCTCACT	–

Genome sequence of *E. coli* MG1655 was used to design the primer sequences because the strain TWF001 [9] was derived from MG1655. The *thr* operon regulatory region was amplified using the genome sequence of MG1655 as template because the sequence of *thr* operon regulatory region in TWF001 was mutated. The insertion fragments were amplified by obtaining the insertion sequence in the first PCR, and then adding the homologous arm in the second PCR. The database on https://salislab.net was used to the design ribosome binding site (RBS) sequence and calculate its translation initiation rate.

### Construction of *E. coli* mutant strains TWF051, TWF052, TWF053, TWF054 and TWF055

**Fig. 2:**
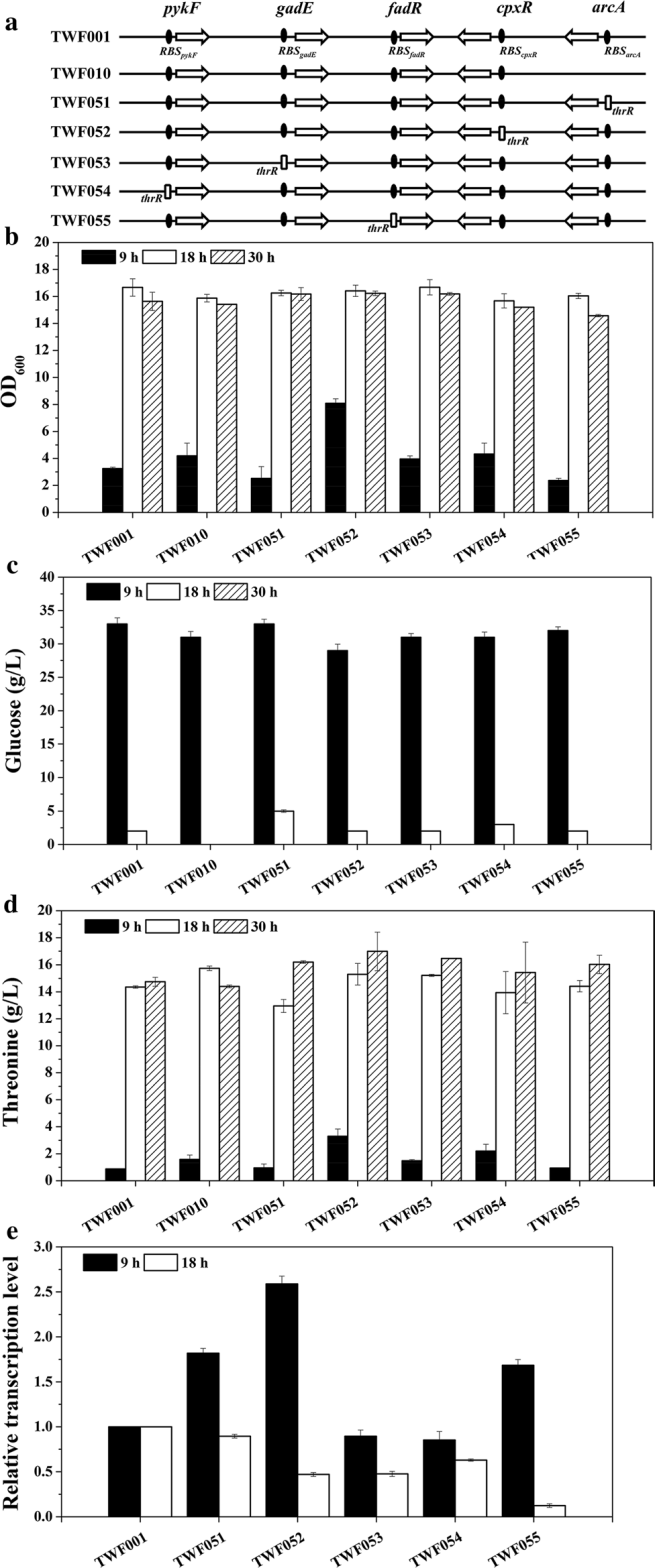
Flask cultivation for l-threonine production in *E. coli* strains TWF001, TWF0010, TWF051, TWF052, TWF053, TWF054 and TWF055. **a** Genetic comparison of the strains constructed by dynamic regulation genes *arcA*, *cpxR*, *gadE*, *pykF* and *fadR*. **b** Cell growth; **c** glucose consumption; **d**l-threonine production; **e** relative transcription levels of *arcA*, *cpxR*, *gadE*, *pykF* and *fadR* in TWF051, TWF052, TWF053, TWF054 and TWF055, respectively. The error bars indicate the standard deviations from three independent experiments

The RBS sequence of the five genes *arcA*, *cpxR*, *gadE*, *pykF* and *fadR* in TWF001 was individually replaced with *thrR* (*RBS*_*thrL*_-*thrL*-*RBS*_*thrA*_, the combined sequence of the *RBS* sequence of *thrL*, *thrL*, and the *RBS* sequence of *thrA*). As shown in Fig. 2a, TWF051 was derived from TWF001 by replacing the native RBS of *arcA* in chromosome with the *thrR*-*arcA* fragments (the *thrR* fragment with the homologous arm of *arcA*), using the CRISPR-Cas9 two-plasmid system [49]. Firstly, the fragments of *thrR*-*arcA* were amplified using the primer pairs P*arcA*-U-FR1/P*arcA*-D-RF1and using the genomic DNA of *E. coli* MG1655 as the template, then amplified using the primer pairs P*arcA*-U-FR11/P*arcA*-D-RF11and using the recovered fragments as a template. Secondly, the plasmid pTargetF-*arcA* was constructed though inverse PCR using the primers Pf-P*arcA*-F/pf-P*arcA*-R and using pTargetF as the template, and then self-ligation; the primers Pf-P*arcA*-V-F/pf-V-R was used to confirm the correctness of the plasmid by PCR. Next, pTargetF-*arcA* and *thrR*-*arcA* were used according to the instruction of the CRISPR–Cas9 system [49]. After 36 h culturing at 30 °C, the correctness of strain was identified using the primer pairs ThrL-V-F/P*arcA*-V-R, and sequenced using the primers P*arcA*-S-F/P*arcA*-S-R. Using the same method, TWF052 was derived from TWF001 by replacing the native RBS of *cpxR* in chromosome with the *thrR*-*cpxR* fragments. The fragment of *thrR*-*cpxR* was amplified using the primer pairs P*cpxR*-U-FR/P*cpxR*-D-RF, and then using the primer pairs P*cpxR*-U-FR11/P*cpxR*-D-RF11. The plasmid pTargetF-*cpxR* was constructed using the primers Pf-P*cpxR*-F/pf-P*cpxR*-R, and the primers Pf-P*cpxR*-V-F/pf-V-R was used to confirm the correctness of the plasmid by PCR. The correctness of strain was identified using the primer pairs ThrL-V-F/P*cpxR*-V-R, and sequenced using the primers P*cpxR*-S-F/P*cpxR*-S-R. TWF053 was derived from TWF001 by replacing the native RBS of *cpxR* in chromosome with the *thrR*-*gadE* fragments. The fragments of *thrR*-*gadE* were amplified using P*gadE*-U-FR/P*gadE*-D-RF, and then using P*gadE*-U-FR11/P*gadE*-D-RF11. The plasmid pTargetF-*gadE* was constructed using the primers Pf-P*gadE*-F/pf-P*gadE*-R and confirmed using the primers Pf-P*gadE*-V-F/pf-V-R. The primer pairs ThrL-V-F/P*gadE*-V-R and the primers P*gadE*-S-F/PgadR-S-R were used to identify and sequence the correctness of strain, respectively. TWF054 was derived from TWF001 by replacing the native RBS of *pykF* in chromosome with the *thrR*-*gadE* fragments. The fragments of *thrR*-*pykF* were amplified using P*pykF*-U-FR/P*pykF*-D-RF, and then using P*pykF*-U-FR11/P*pykF*-D-RF11. The plasmid pTargetF-*pykF* was constructed using the primers Pf-P*pykF*-F/pf-P*pykF*-R and confirmed using the primers Pf-P*pykF*-V-F/pf-V-R. The primer pairs ThrL-V-F/P*pykF*-V-R and the primers P*pykF*-S-F/P*pykF*-S-R were used to identify and sequence the correctness of strain. TWF055 was derived from TWF001 by replacing the native RBS of *fadR* in chromosome with the *thrR*-*fadR* fragments. The fragments of *thrR*-*fadR* were amplified using P*fadR*-U-FR/P*fadR*-D-RF, and then using P*fadR*-U-FR11/P*fadR*-D-RF11. The plasmid pTargetF-*fadR* was constructed using the primers Pf-P*fadR*-F/pf-P*fadR*-R and confirmed using the primers Pf-P*fadR*-V-F/pf-V-R. The primer pairs ThrL-V-F/P*fadR*-V-R and the primers P*fadR*-S-F/P*fadR*-S-R were used to identify and sequence the correctness of strain, respectively.

### Construction of *E. coli* mutant strains TWF056, TWF057, TWF058, TWF059, TWF060, TWF061, TWF062, TWF063, TWF064 and TWF065

**Fig. 3:**
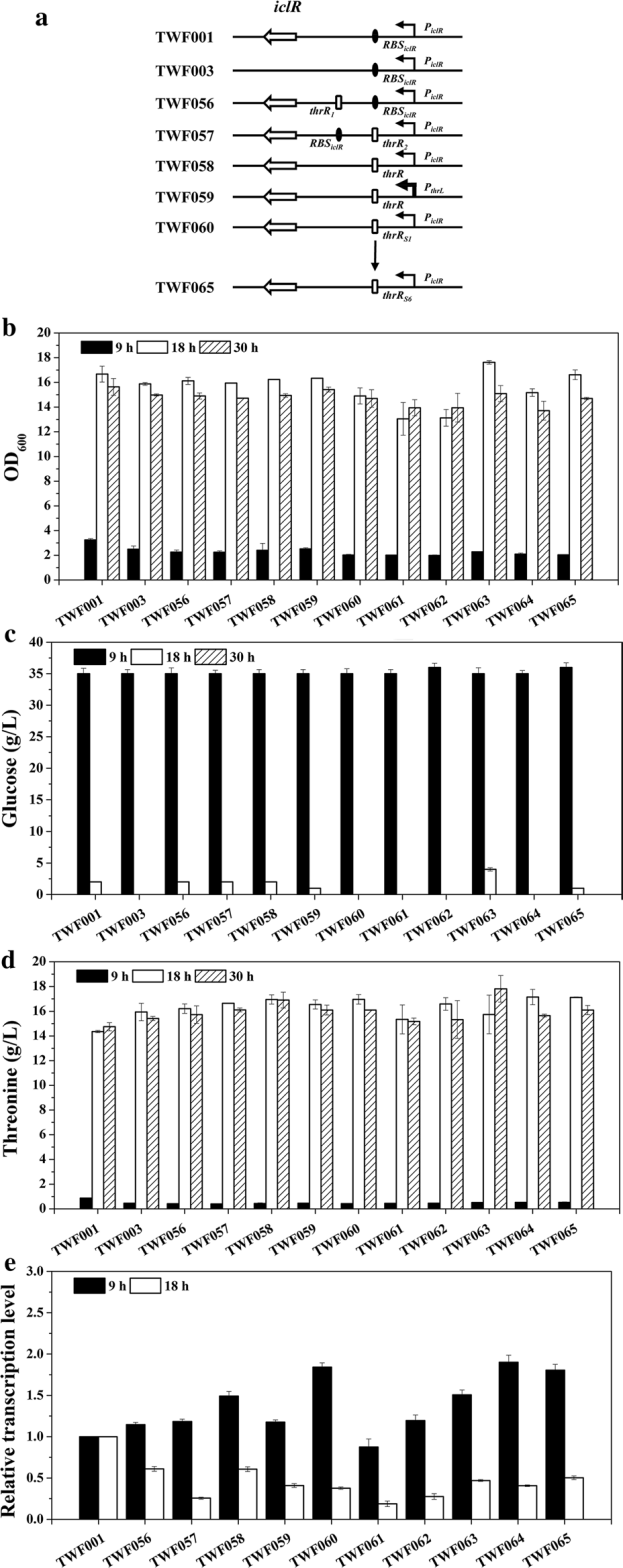
Flask cultivation for l-threonine production in *E. coli* strains TWF001, TWF003, TWF056, TWF057, TWF058, TWF059, TWF060, TWF061, TWF062, TWF063, TWF064, TWF065. **a** Genetic comparison of the strains constructed by dynamic regulation genes *iclR*. **b** Cell growth; **c** glucose consumption; **d**l-threonine production; **e** relative transcription levels of *iclR*. The error bars indicate the standard deviations from three independent experiments

As shown in Fig. 3a, TWF056 was derived from TWF001 by inserting the *thrL*-*RBS*_*thrA*_ (*thrR*_*1*_) fragment between the native ribosomal binding site of the *iclR* (*RBS*_*iclR*_) and the *iclR* sequence, using the CRISPR–Cas9 two-plasmid system [49]. Firstly, the *thrR*_*1*_ fragment was amplified using the primer pairs P*iclR*-U-FR1/P*iclR*-D-RF1 and using the genomic DNA of MG1655 as a template, and then amplified using the primer pairs P*iclR*-U-FR11/P*iclR*-D-RF1 and using the recovered fragments as a template. Secondly, the plasmid pTargetF-*iclR1* was constructed using the primers Pf-P*iclR*-F/pf-P*iclR*-R by inverse PCR using pTargetF as template, and then self-ligation. The primers Pf-P*iclR*-V-F/pf-V-R was using to confirm the correctness of the plasmid by PCR. The primer pairs ThrL-V-F/P*iclR*-V-R and the primers P*iclR*-S-F/P*iclR*-S-R were used to identify and sequence the correctness of strain, respectively. Using the same method, TWF057 was constructed. *RBS*_*thrL*_-*thrL* (*thrR*_*2*_) was amplified using the primers P*iclR*-U-FR2/P*iclR*-D-RF2, and then amplified using the P*iclR*-U-FR22/P*iclR*-D-RF22. The plasmid pTargetF-*iclR2* was constructed using the primers Pf-P*iclR*-F2/pf-P*iclR*-R2, and identified using the primers Pf-P*iclR*-V-F2/pf-V-R). To construct TWF058, the sequence *RBS*_*thrL*_-*thrL*-*RBS*_*thrA*_ (*thrR*) was amplified using P*iclR*-U-FR2/P*iclR*-D-RF1, and then amplified using P*iclR*-U-FR22/P*iclR*-D-RF11. The plasmid pTargetF-*iclR1* was used. To constructed TWF059, the sequence *P*_*thrL*_-*thrR* was amplified using P*iclR*-U-FR3/P*iclR*-D-RF1, and then amplified using P*iclR*-U-FR33/P*iclR*-D-RF11. The plasmid pTargetF-*iclR1* was used. To construct TWF060, the sequence *RBS*_*thrL*_-*thrL*-*RBS*_*s1*_ (*thrR*_*s1*_) was amplified using P*iclR*-U-FR2/P*iclR*-D-RF4, and then amplified using P*iclR*-U-FR22/P*iclR*-D-RF44. The plasmid pTargetF-*iclR1* was used. To construct TWF061, the sequence *RBS*_*s2*_-*thrL*-*RBS*_*thrA*_ (*thrR*_*s2*_) was amplified using P*iclR*-U-FR5/P*iclR*-D-RF4, and then amplified using P*iclR*-U-FR55/P*iclR*-D-RF44. The plasmid pTargetF-*iclR1* was used. To construct TWF062, the sequence *RBS*_*s3*_-*thrL*-*RBS*_*thrA*_ (*thrR*_*s3*_) was amplified using P*iclR*-U-FR6/P*iclR*-D-RF4, and then amplified using P*iclR*-U-FR55/P*iclR*-D-RF44. The plasmid pTargetF-*iclR1* was used. To construct TWF063, the sequence *RBS*_*s4*_-*thrL*-*RBS*_*thrA*_ (*thrR*_*s4*_) was amplified using P*iclR*-U-FR7/P*iclR*-D-RF4, and then amplified using P*iclR*-U-FR55/P*iclR*-D-RF44. The plasmid pTargetF-*iclR1* was used. To construct TWF064, the sequence *RBS*_*s5*_-*thrL*-*RBS*_*thrA*_ (*thrR*_*s5*_) was amplified using P*iclR*-U-FR8/P*iclR*-D-RF4, and then amplified using P*iclR*-U-FR55/P*iclR*-D-RF44. The plasmid pTargetF-*iclR1* was used. To construct TWF065, the sequence *RBS*_*s6*_-*thrL*-*RBS*_*thrA*_ (*thrR*_*s6*_) was amplified using P*iclR*-U-FR9/P*iclR*-D-RF4, and then amplified using P*iclR*-U-FR55/P*iclR*-D-RF44. The plasmid pTargetF-*iclR1* was used.

### Construction of *E. coli* mutant strains TWF066, TWF067, TWF068, TWF069, TWF070, TWF071, TWF072, TWF073, TWF074, TWF075, TWF076, TWF077, TWF078, TWF079, TWF080, TWF081, TWF082 and TWF083

**Fig. 4:**
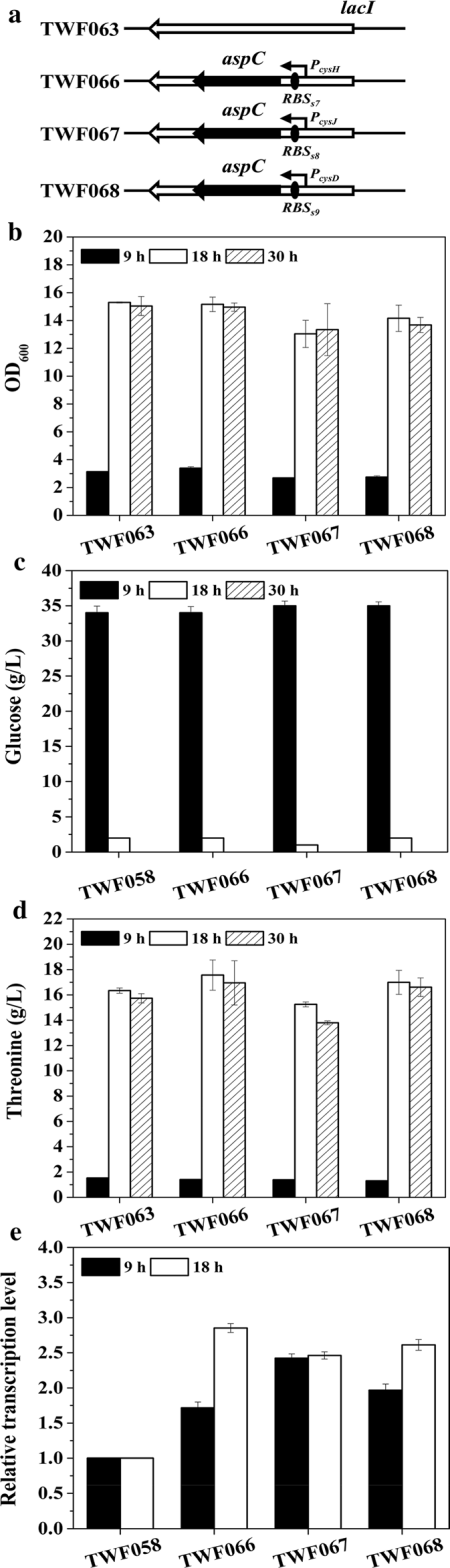
Flask cultivation for l-threonine production in *E. coli* strains TWF063, TWF066, TWF067 and TWF068. **a** Genetic comparison of the strains constructed by dynamic regulation gene *aspC*. **b** Cell growth; **c** glucose consumption; **d**l-threonine production; **e** relative transcription levels of *aspC*. The error bars indicate the standard deviations from three independent experiments

As shown in Fig. 4a, TWF066 was derived from TWF063 by inserting the gene *aspC* controlled by the promoter of *cysH* (*P*_*cysH*_) and *RBS*_*s7*_ into the *lacI* locus in chromosome. The fragment of *RBS*_*s7*_-*aspC* was amplified by using the primers *cysH*-*aspC*-F2/*cysH*-*aspC*-R2 and using the genomic DNA of TWF001 as a template. The promoter of *cysH* was amplified by using the primers *cysH*-*aspC*-F1/*cysH*-*aspC*-R1 and using the genomic DNA of TWF001 as template. These two DNA fragments were linked together by overlap PCR, using the primers cys-*aspC*-F/cys-*aspC*-R, and resulting in the replacement fragment *P*_*cysH*_-*RBS*_*s7*_-*aspC*. The plasmid pTargetF-*lacI* was constructed using Pf-*lacI*-F/pf-*lacI*-R, and confirmed using Pf-*lacI*-V-F/pf-V-R. The primers *lacI*-aspC-V-F/*lacI*-aspC-V-R and *lacI*-S-F/*lacI*-S-R were used to identify and sequence the correctness of the strain, respectively. TWF067 was constructed from TWF001 by inserting the gene *aspC* controlled by the promoter of *cysJ* (*P*_*cysJ*_) and *RBS*_*s8*_ into the *lacI* locus in chromosome. The fragment of *RBS*_*s8*_-*aspC* was amplified by using the primers *cysJ*-*aspC*-F2/*cysH*-*aspC*-R2, and using the genomic DNA of TWF001 as a template. The promoter of *cysJ* was amplified by using the primers *cysJ*-*aspC*-F1/*cysJ*-*aspC*-R1, and using the genomic DNA of TWF001 as a template. These two DNA fragments were linked together by overlap PCR, using the primers cys-*aspC*-F/cys-*aspC*-R, resulting in the replacement fragment *P*_*cysJ*_-*RBS*_*s8*_-*aspC*. TWF068 was constructed from TWF001 by inserting the gene *aspC* controlled by the promoter of *cysD* (*P*_*cysD*_) and *RBS*_*s9*_ into the *lacI* locus in chromosome. The fragment of *RBS*_*s9*_-*aspC* was amplified by using the primers *cysD*-*aspC*-F2/*cysH*-*aspC*-R2, and using the genomic DNA of TWF001 as a template. The promoter of *cysD* was amplified by using the primers *cysD*-*aspC*-F1/*cysD*-*aspC*-R1, using the genomic DNA of TWF001 as a template. These two DNA fragments were linked together by overlap PCR, using the primers cys-*aspC*-F/cys-*aspC*-R, resulting in the replacement fragment *P*_*cysD*_-*RBS*_*s9*_-*aspC*.

**Fig. 5:**
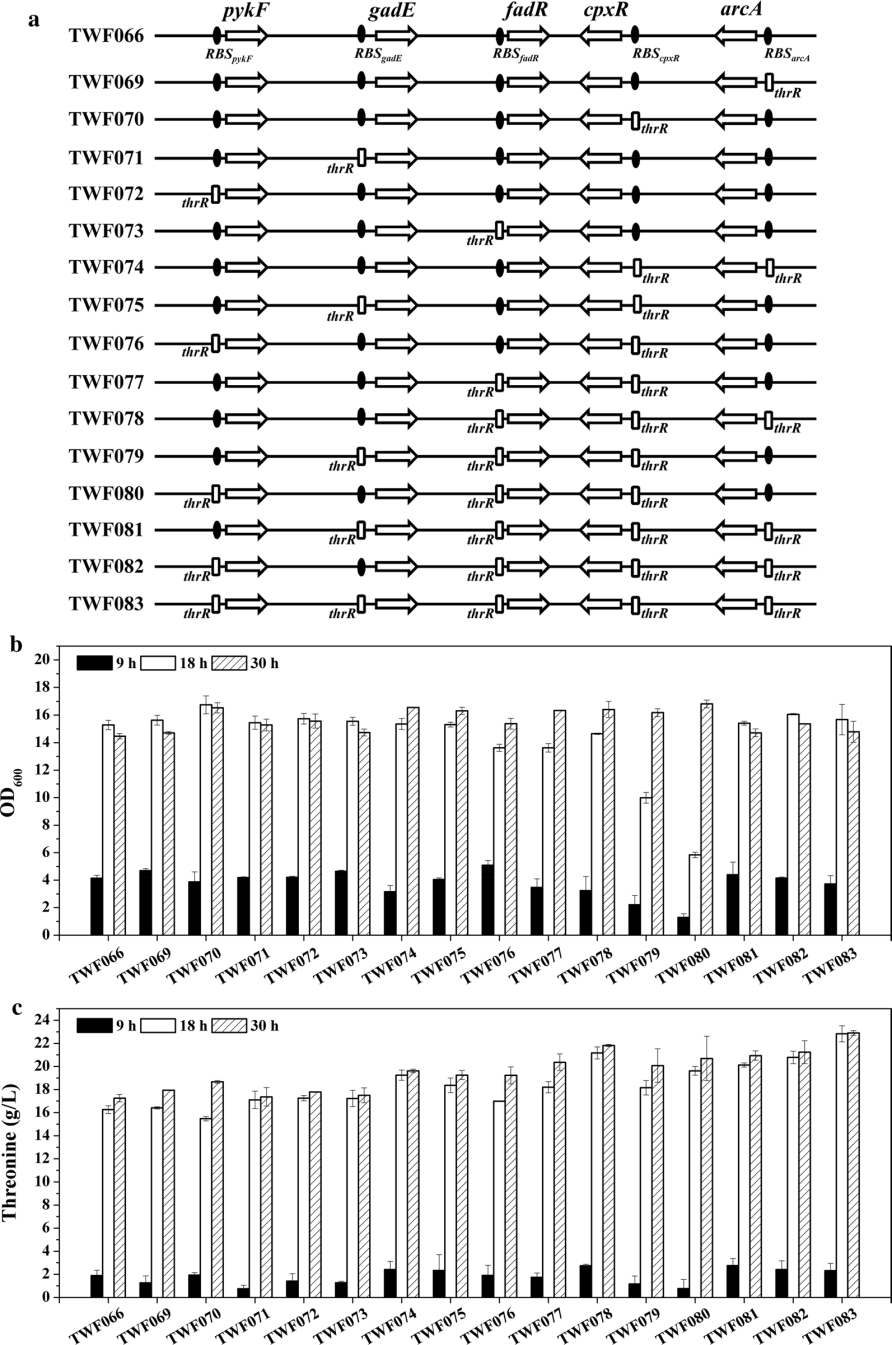
Flask cultivation for l-threonine production in *E. coli* strains TWF066, TWF069, TWF070, TWF071, TWF072, TWF073, TWF074, TWF075, TWF076, TWF077, TWF078, TWF079, TWF080, TWF081, TWF082 and TWF083. **a** Genetic comparison of the strains constructed by dynamic regulation genes *arcA*, *cpxR*, *gadE*, *pykF* and *fadR*. **b** Cell growth; **c**l-threonine production. The error bars indicate the standard deviations from three independent experiments

As shown in Fig. 5a, TWF069, TWF070, TWF071, TWF072 and TWF073 were derived from TWF066 using the same method to construct TWF051, TWF052, TWF053, TWF054 and TWF055, respectively. TWF074, TWF075, TWF076 and TWF077 were derived from TWF070 using the same method to construct TWF051, TWF053, TWF054 and TWF055, respectively. TWF078, TWF079 and TWF080 were derived from TWF077 using the same method to construct TWF051, TWF053 and TWF054, respectively. TWF081 and TWF082 were derived from TWF078 using the same method to construct TWF053 and TWF054, respectively. TWF083 was derived from TWF081 using the same method to construct TWF054.

### Flask fermentation and fed-batch fermentation

*Escherichia coli* strains were streaked from the frozen stock on LB plate and cultured at 37 °C overnight. Then, a loop of bacteria was transferred into a tube containing 5 mL LB medium and cultured at 37 °C for 5 h with 200 rpm shaking. Next, the culture was transferred into a 250-mL flask containing 30 mL LB medium (the initial OD_600_ of 0.05) and cultured at the same growth condition for 6 h. Finally, 5 mL culture was transferred into a 500-mL flask containing 30 mL fermentation medium (2 g/L yeast extract, 2 g/L citric acid, 25 g/L (NH_4_)_2_SO_4_, 7.46 g/L KH_2_PO_4_, 40 g/L glucose, 2 g/L MgSO_4_·7 H_2_O, 5 mg/L FeSO_4_·7H_2_O, 5 mg/L MnSO_4_·4 H_2_O, and 20 g/L CaCO_3_, pH 6.8) [50] at 37 °C with 200 rpm shaking for flask fermentation, or the whole culture was transferred into a quadruple bioreactor (Parallel-Bioreactor, China) containing 1 L fermentation medium (30 g/L glucose, 20 g/L (NH_4_)_2_SO_4_, 3 g/L yeast extract, 2 g/L KH_2_PO_4_, 2 g/L MgSO_4_·7H_2_O, 5 mg/L FeSO_4_·7H_2_O, 5 mg/L MnSO_4_·4 H_2_O) [6]. Temperature was maintained at 37 °C, the aeration rate at 1.5 vvm, pH was maintained automatically at 6.9 with NH_4_OH, and the dissolved oxygen value was maintained below 30%. Biomass was characterized by the OD_600_ value. One unit of OD_600_ corresponds to 1.7 g/L cell wet weight [6]. The amount of amino acids was determined by the 1200 series HPLC system (Agilent Technology, USA), using the orthophthalaldehyde precolumn derivatization method [51].

Acetate, pyruvate and oxaloacetate were quantified by using 1200 Series HPLC system (Agilent Technology, USA) equipped with an amines HPX-87H column (300 × 7.8 mm), and 0.005 M H_2_SO_4_ was used as a mobile phase with a flow rate of 0.5 mL/Min. The column temperature was maintained at 40 °orthophthalaldehyde and the UV absorption was determined at 210 nm.

### Quantification of mRNA

The transcriptional levels of *iclR*, *arcA*, *cpxR*, *gadE*, *pykF*, *fadR* and *aspC* in different *E. coli* strains was quantified using the real-time PCR (RT-PCR). Total RNA was extracted from different *E. coli* cells grown at 9 h and 18 h. According to the published method [52, 53], the relative abundance of the targeted mRNAs was quantified based on the cycle threshold value, which is defined as the number of cycles required to obtain a fluorescent signal above the background and was calculated. The relative abundance of 16S rRNA was used as an internal standard. All assays were performed in triplicate.

### Statistical analysis

Experimental data were expressed as means ± standard deviations. Statistical comparisons were made by one-way ANOVA to detect significant difference for l‐threonine production and glucose consumption between control and experimental groups. Spots with an adjusted p-value<0.05 were considered statistically significant.

## Results

### Expression regulation of *arcA*, *cpxR*, *gadE*, *pykF* and *fadR* to increase l-threonine production in *E. coli* TWF001

The *thr* operon controls the expression of l-threonine synthesis in *E. coli* by *thrL* [11]. ArcA represses some genes involved in TCA cycle and glyoxylate shunt [34], and l-threonine production increased in the *arcA* mutant TWF010 [46]. Pyruvate kinase encoded by *pykF* is important for cellular metabolism and glycolysis in *E. coli*, and the *pykF* mutant THRD produced more l-threonine [48]. FadR regulates the transcription of most genes involving in fatty acid degradation [28], and the mutant TWF044 in which both *iclR* and *fadR* were deleted could increase l-threonine production [6]. CpxR and GadE can activate the genes involved in the type II fatty acid synthase systems [32, 33]. Since the genes *arcA*, *cpxR*, *gadE*, *pykF* and *fadR* are important for l-threonine biosynthesis in *E. coli*, it is necessary to dynamically regulate their expression. In this study, the RBS sequence of the five genes *arcA*, *cpxR*, *gadE*, *pykF* and *fadR* in TWF001 was individually replaced with *thrR*, resulting in the stains TWF051, TWF052, TWF053, TWF054 and TWF055, respectively (Fig. 2a).

After 30 h flask fermentation, the maximum OD_600_ of *E. coli* TWF001 reached 16.67 and produced 14.75 g/L l-threonine from 40 g/L glucose (Fig. 2). Compared with TWF001, the maximum OD_600_ (15.88) of *arcA* deletion mutant TWF010 [46] decreased, but the maximum l-threonine production of TWF010 increased (Fig. 2). TWF010 consumed glucose faster than TWF001 and other five mutant strains, and used up all the glucose after 18 h; after 30 h, glucose was completely consumed in all strains (Fig. 2c).

The maximum l-threonine productions in TWF051, TWF052, TWF053, TWF054 and TWF055 were all lager than TWF001 and TWF010 (Fig. 2d). This indicates that the expression regulation of these five genes is beneficial for l-threonine biosynthesis in *E. coli*.

The transcriptional levels of *arcA* in TWF051, *cpxR* in TWF052, *gadE* in TWF053, *pykF* in TWF054, and *fadR* in TWF055 were determined (Fig. 2e). All these genes were up-regulated in the early stage of fermentation (9 h), but down-regulated in the later stage (18 h). This indicates that the dynamic regulation of the target gene worked in all five mutants and explains the higher l-threonine production in these mutants, although the degree of regulation varies with the target gene. The maximum l-threonine production (16.99 g/L) was obtained in TWF052 with a yield of 0.424 g/g glucose.

### Expression regulation of *iclR* to increase l-threonine production in *E. coli* TWF001

In previous work, we have developed an l-threonine-producing strain TWF003 from *E. coli* TWF001 by deleting *iclR*. The l-threonine production in TWF003 reached 11.76 g/L, which is a 26% increase compared to TWF001 [9]. Deleting *fadR* [6] or *arcA* [46] in TWF003 could further increase l-threonine production. Since IclR regulates the expression of the *aceBAK* operon which could induce the glyoxylate bypass, and this could bypass prevent the quantitative loss of the entering carbon as CO_2_ in the Krebs cycle [54], the deletion of *iclR* might influence the cell growth of *E. coli*; therefore, dynamically regulating the expression of *iclR* should be a good strategy to balance the cell growth and l-threonine production in TWF001. Since IclR also regulates its own expression, we tried to change its regulatory region to regulate the expression of *iclR* according to the intracellular l-threonine concentration. Starting from TWF001, TWF056 was constructed by inserting *thrR*_*1*_ between *RBS*_*iclR*_ and *iclR*, TWF057 was constructed by inserting *thrR*_*2*_ between *P*_*iclR*_ and *RBS*_*iclR*_, TWF058 was constructed by replacing *RBS*_*iclR*_ with *thrR*, and TWF059 was constructed by replacing *P*_*iclR*_ and *RBS*_*iclR*_ with *P*_*thrl*_-*thrR* (Fig. 3a).

During flask fermentation, the maximum OD_600_ of TWF056, TWF057, TWF058 and TWF059 reached 16.12, 15.94, 16.24 and 16.34, respectively, which are higher than that of TWF001 but lower than that of TWF003 [9] (Fig. 3b). The results indicate that the autoregulation of *iclR* expression in these mutants did not significantly affect the cell growth.

Glucose consumption patterns are quite different in these strains. Similar levels of glucose were consumed in these strains after 9 h. After 18 h glucose was completely consumed in TWF003, TWF060, TWF061, TWF062, and TWF064. After 30 h, glucose was used up in all strains (Fig. 3c). During flask fermentation, TWF056, TWF057, TWF058 and TWF059 produced more l-threonine than TWF001 and TWF003 (Fig. 3d). The results indicate that dynamic regulation of *iclR* expression is better than deleting *iclR* to improve l-threonine biosynthesis in *E. coli*. Among these four strains, TWF058 produced the highest amount of l-threonine (16.95 g/L) after 18 h flask cultivation, which is 6.34% more than that of TWF003. This suggests that the dynamic regulatory *iclR* expression by replacing *RBS*_*iclR*_ with *thrR* is a good choice for improving l-threonine production in *E. coli*.

To understand the difference on growth rate and l-threonine productions in the above mutant strains, the transcriptional level of *iclR* were determined by RT-PCR (Fig. 3e). Compared to the control TWF001, the transcriptional level of *iclR* in all four mutant strains increased after 9 h but decreased after 18 h. This suggests that the expression of *iclR* in these strains were dynamically regulated, i.e., the expression of *iclR* was up-regulated in the early stage of the growth but down-regulated in the late stage of the growth.

In order to optimize the dynamic regulation of *iclR* expression, different RBS in *thrR* were designed (Table 4). The mutants TWF060, TWF061, TWF062, TWF063, TWF064 and TWF065 were constructed from TWF058 by replacing *RBS*_*iclR*_ with *thrR*_*s1*_, *thrR*_*s2*_, *thrR*_*s3*_, *thrR*_*s4*_, *thrR*_*s5*_, and *thrR*_*s6*_, respectively (Table 1 and Fig. 3a). After 30 h cultivation, the maximum OD_600_ of TWF060, TWF061, TWF062, TWF063, TWF064 and TWF065 reached 14.90, 13.95, 13.95, 17.62, 15.17 and 16.62, respectively; the maximum production of l-threonine in TWF060, TWF061, TWF062, TWF063, TWF064 and TWF065 reached 16.96, 15.34, 16.58, 17.81, 17.15 and 17.12, respectively. Among these mutants, TWF063is the best according to the growth rate and l-threonine productions.

The transcriptional levels of *iclR* in TWF060, TWF061, TWF062, TWF063, TWF064 and TWF065 were also determined by RT-PCR, using TWF001 as the control. Compared to TWF001, the transcription levels of *iclR* in TWF061, TWF062, TWF063, TWF064 and TWF065 were up-regulated in the early stage of the growth (9 h) but down-regulated in the late stage (18 h). This indicates again that the regulation of *iclR* in these mutants satisfies the growth needs in the early stage of fermentation as well as the needs for l-threonine production in the late stage of fermentation. The maximum l-threonine production (17.81 g/L) was obtained in TWF063 with a yield of 0.445 g/g glucose.

### Expression regulation of *aspC* to improve l-threonine production in *E. coli* TWF063

Previously, l-threonine producing *E. coli* strain TWF006 was constructed from TWF004 by inserting the *trc* promoter in the upstream of *aspC* in chromosome [9]. The l-threonine production in TWF006 reached 12.47 g/L from 30 g/L glucose after 36 h flask fermentation, which is 11.6% increase compared to the control TWF004 [9]. More l-threonine was also produced when *P*_*tac*_-*aspC* was inserted into the *lacZ* locus of *E. coli* TWF041 [6]. Since the expression regulation of *aspC* is related to the cell morphology and growth [55], the dynamic expression regulation of *aspC* is necessary to improve l-threonine production in *E. coli*. Therefore, the promoters of *cysH*, *cysJ* and *cysD* which can be activated by l-threonine [12] were fused with different RBS sequences to dynamically regulate the l-threonine production in TWF063. The fragments *P*_*cysH*_-*RBS*_*s7*_-*aspC*, *P*_*cysJ*_-*RBS*_*s8*_-*aspC* or *P*_*cysD*_-*RBS*_*s9*_-*aspC* were individually inserted into the *lacI* loci in TWF063, resulting in strains TWF066, TWF067 and TWF068, respectively (Fig. 4a).

Glucose consumption patterns are quite similar in these strains. After 30 h, glucose was used up in all strains (Fig. 4c). After 30 h flask fermentation, TWF063 produced 16.34 g/L l-threonine from 40 g/L glucose and its maximum OD_600_ reached 15.30 with glucose being completely consumed (Fig. 4). TWF066 grew (15.16) similar with TWF063 but produced more l-threonine (17.56 g/L) than TWF063. TWF067 and TWF068 grew slower than TWF063, and TWF067 produced less l-threonine (15.25 g/L) than TWF063, but TWF068 produced more l-threonine (17.00 g/L) than TWF063. The results indicate that the regulation of these three promoters was different, and different expression of *aspC* could affect the growth and l-threonine production. To understand the difference on growth and l-threonine productions in the above mutant strains, the transcriptional level of *apsC* was determined by RT-PCR (Fig. 4e). Compared to the control TWF063, the transcriptional level of *aspC* was increased in all three mutant strains during fermentation. At 9 h, the expressions of *aspC* in TWF066 and TWF068 were lower than that in TWF067, while at 18 h, the expression of *aspC* in TWF066 and TWF068 was higher than that in TWF067. Among the three mutants, the expression difference of *aspC* between 9 h and 18 h in TWF066 was the largest, which is consistent with the highest production of l-threonine in TWF066 This suggests that the promoter of *cysH* is most sensitive to l-threonine among the three promoters. The maximum l-threonine production (17.56 g/L) was obtained in TWF066 with a yield of 0.439 g/g glucose.

### Expression of multiple genes to improve l-threonine production in *E. coli*

Since dynamic regulation of *arcA*, *cpxR*, *gadE*, *fadR* or *pykF* could increase l-threonine production in TWF001 (Fig. 2), these genes were also designed to be dynamically regulated in TWF066, resulting in strains TWF069, TWF070, TWF071, TWF072 and TWF073 (Fig. 5a).

After 30 h flask fermentation, TWF066 can produce 17.35 g/L l-threonine from 40 g/L glucose and its maximum OD_600_ reached 15.29 with glucose being completely consumed. The maximum OD_600_ of TWF069, TWF070, TWF071, TWF072 and TWF073 reached 15.63, 16.75, 15.46, 15.74 and 15.55, respectively. TWF069, TWF070, TWF071, TWF072 and TWF073 produced 17.94, 18.66, 17.37, 17.78 and 17.51 g/L l-threonine, respectively. Among the five mutants, TWF070 is the best based on cells growth and l-threonine yield (Fig. 5).

Mutant strains TWF074 (regulating *arcA*), TWF075 (regulating *gadE*), TWF076 (regulating *pykF*) and TWF077 (regulating *fadR*) was constructed from TWF070 (Fig. 5a). The maximum OD_600_ of TWF074, TWF075, TWF076 and TWF077 reached 16.56, 16.32, 15.38 and 16.34, respectively. TWF074, TWF075, TWF076 and TWF077 produced 19.61, 19.25, 19.23 and 20.36 g/L l-threonine, respectively. Among the four mutants, TWF077 produced the highest l-threonine yield.

Mutant strains TWF078 (regulating *arcA*), TWF079 (regulating *gadE*) and TWF080 (regulating *pykF*) was constructed from TWF077. The maximum OD_600_ of TWF078, TWF079 and TWF080 reached 16.41, 16.19 and 16.82, respectively (Fig. 5b). TWF078 and TWF080 produced more l-threonine (21.81 and 20.70 g/L, respectively) than TWF077.

From TWF078, mutant strains TWF081 (regulating *gadE*) and TWF082 (regulating *pykF*) was constructed. From TWF081, mutant strain TWF083 (regulating *pykF*) was further constructed (Fig. 5a). The maximum OD_600_ of TWF081, TWF082 and TWF083 reached 15.42, 16.07 and 15.68, respectively. TWF081 and TWF082 produced less l-threonine (20.94 and 21.24 g/L, respectively) than TWF078, but TWF083 produced more l-threonine (22.90 g/L) than TWF078. Among all the mutants, TWF083 produced the highest l-threonine yield.

### Comparison of some key l-threonine producing *E. coli* strains

**Fig. 6:**
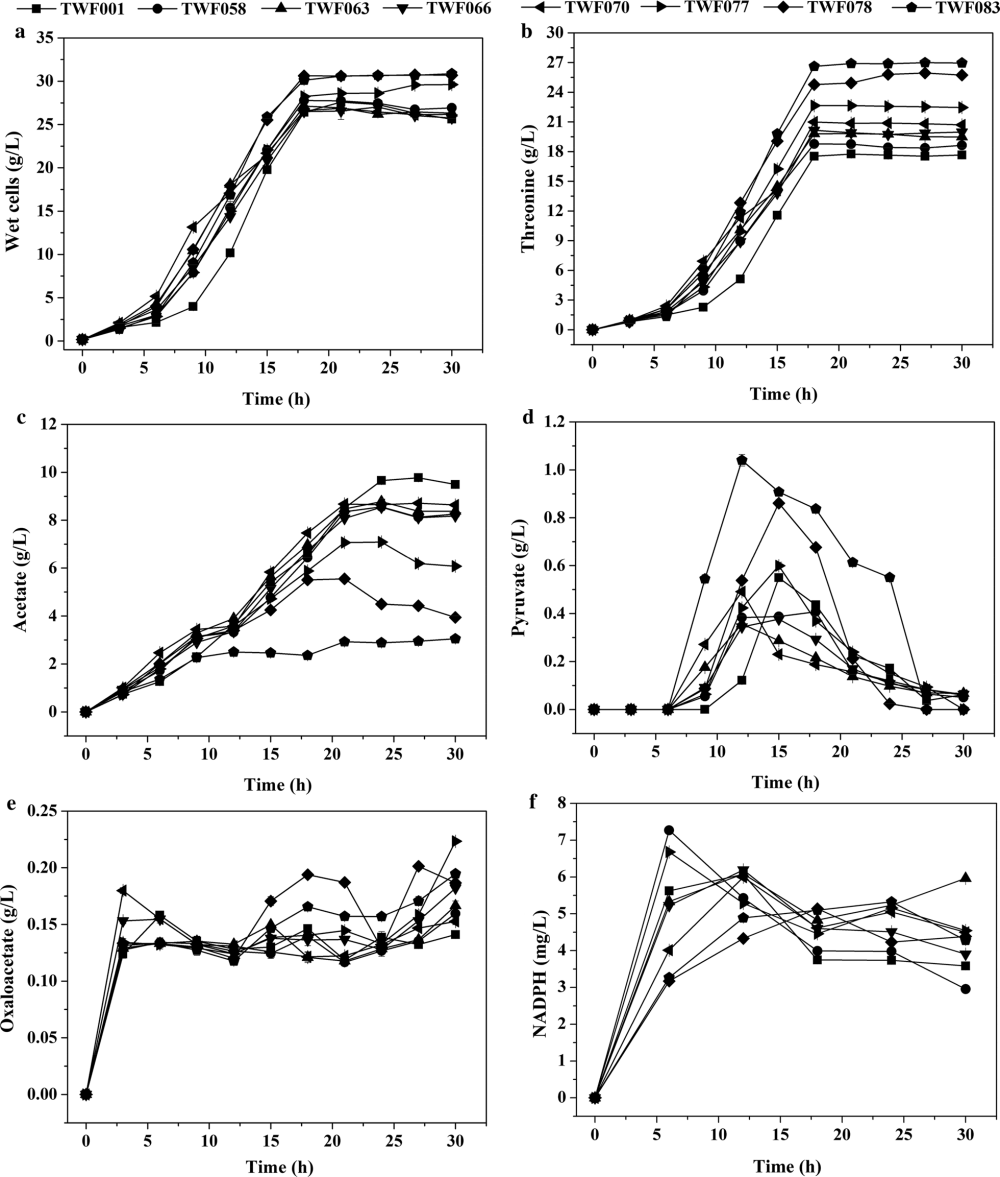
Comparison of cell growth (**a**), l-threonine production (**b**), acetate concentration (**c**), pyruvate concentration (**d**), oxaloacetate concentration (**e**), and NADPH level (**f**) during flask fermentation of TWF001, TWF058, TWF063, TWF066, TWF070, TWF077, TWF078 and TWF083. The error bars indicate the standard deviations from three independent experiments

To compare their high l-threonine production, flask fermentations of some key mutant strains were performed (Fig. 6). Compared with the control TWF001 [56], all mutant strains produced more l-threonine, and TWF083 produced the most (Fig. 6b). The highest specific growth rate was observed around 3 h for all strains, and then the specific growth rate decreased with time (Fig. 6a). The highest specific l-threonine production rate was observed between 9 and 15 h in these strains (Fig. 6b). During 6 h to 15 h, the specific l-threonine production rate of TWF078 was higher than TWF083, but the final production of l-threonine was lower than TWF083; possibly because that the l-threonine production of TWF078 (0.774 g/L) was much lower than that of TWF083 (0.813 g/L) at 3 h. The acetate concentration in the mutant strains during the fermentation was quite different from that of TWF001, and much less acetate was accumulated in TWF083 (Fig. 6c). The pyruvate concentration in the mutant strains during the fermentation was also quite different from that of TWF001 (Fig. 6d). TWF083 and TWF078 produced more pyruvate than other strains during 6–24 h. This data indicates that in the mutant strains, especially TWF083, more carbon source was used for l-threonine production. TWF083 and TWF078 also produced more oxaloacetate, the precursor for l-threonine synthesis, than other strains during the fermentation (Fig. 6e). The intracellular NADPH content of TWF078 and TWF083 were lower than that of other strains before 18 h, suggesting that they consumed more NADPH for l-threonine biosynthesis (Fig. 6f).

### Effect of glucose concentration on l-threonine production in *E. coli* TWF083

**Fig. 7:**
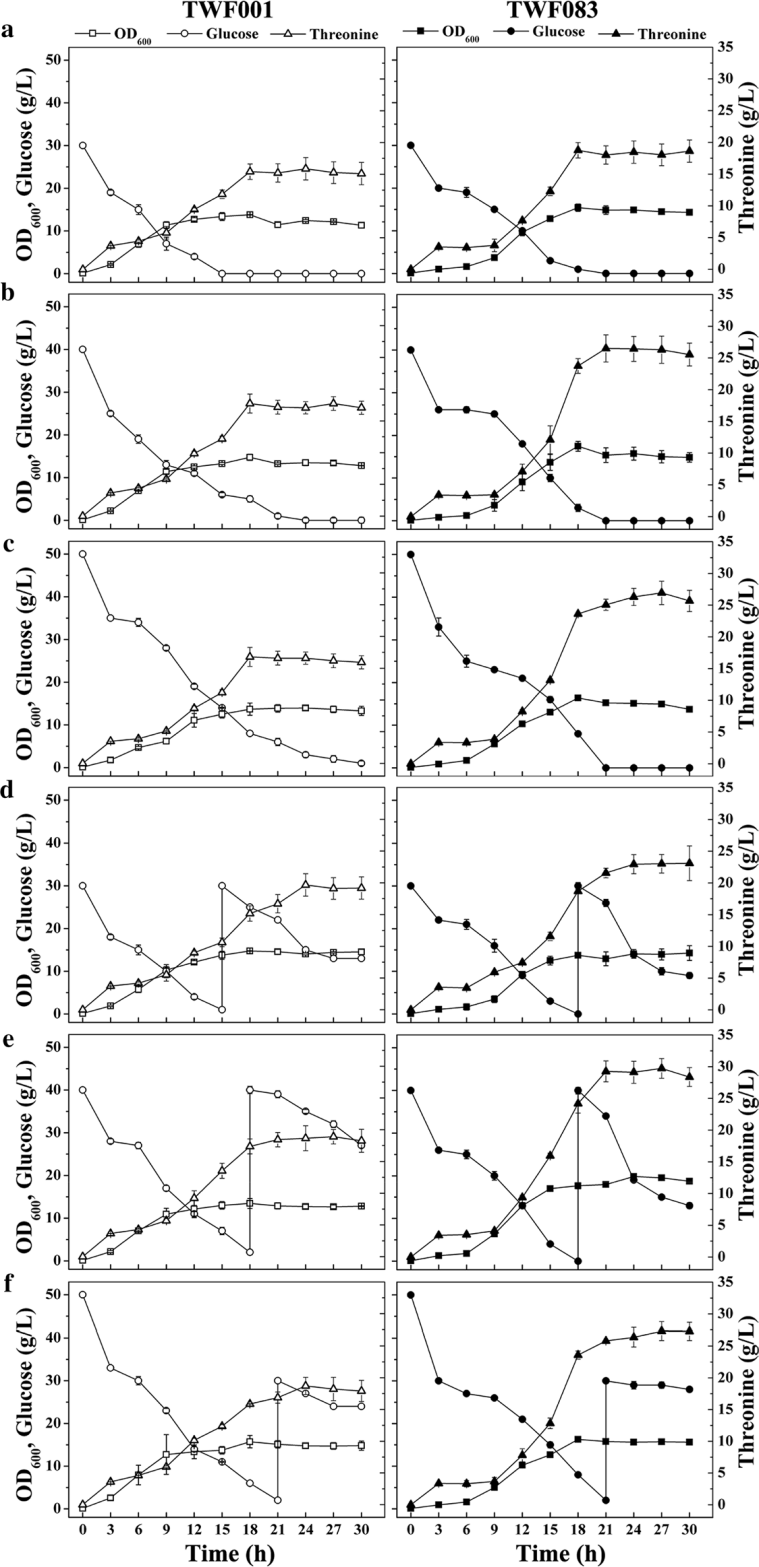
Comparison for l-threonine production in *E. coli* strains TWF001 and TWF083 under different glucose addition. **a** 30 g/L glucose; **b** 40 g/L glucose; **c** 50 g/L glucose; **d** 2 * 30 g/L glucose, the first was added at the beginning and the second when the glucose was used up completely, respectively; **e** 2 * 40 g/L glucose, the first was added at the beginning and the second at 18 h, respectively; **f** 50 and 30 g/L glucose, the first was added at the beginning and the second at 21 h, respectively. The error bars indicate the standard deviations from three independent experiments

TWF001 and TWF083 were grown in medium with different glucose concentrations (Fig. 7). When the initial glucose concentration in the medium increased from 30 to 50 g/L, the cell growth pattern of TWF001 changed. when grown in medium containing 30, 40, and 50 g/L initial glucose, glucose in TWF001 was completely consumed after 15, 24 and 30 h, and the maximum OD_600_ of TWF001 reached 13.80, 14.76, and 13.96, respectively (Fig. 7a–c). The cell growth and glucose consumption patterns of TWF083 were quite different from TWF001 (Fig. 7). When the initial glucose was 30 g/L, the glucose was depleted after 18 h, and the highest OD_600_ reached 15.44. When the initial glucose was 40 g/L, the glucose was depleted after 21 h, and the highest OD_600_ reached 17.44 When the initial glucose was 50 g/L, the glucose was depleted after 21 h, and the highest OD_600_ reached 16.36).

l-Threonine productions in TWF001 and TWF083 grown in medium containing with different concentrations of glucose are also shown in Fig. 7. Under the same growth conditions, TWF083 always produced more l-threonine than TWF001. The highest l-threonine production was obtained in either TWF001 or TWF083 when grown in medium containing 40 g/L initial glucose. When grown in medium with 30 g/L initial glucose for 18 h, TWF083 produced 18.76 g/L l-threonine, while TWF001 produced 15.86 g/L l-threonine. When grown in medium with 40 g/L initial glucose for 21 h, TWF083 produced 26.50 g/L l-threonine, and the conversion rate of glucose to l-threonine reached 0.66 g/g, while TWF001 produced 17.74 g/L l-threonine, and the conversion rate of glucose to l-threonine reached 0.44 g/g. When grown in medium with 50 g/L initial glucose for 27 h, TWF083 produced 26.93 g/L l-threonine, while TWF001 produced 16.58 g/L l-threonine. The results suggest that TWF083 could efficiently consume glucose to produce l-threonine.

When TWF083 grown in the medium containing 40 g/L glucose for 21 h, glucose was depleted, and l-threonine production reached the highest (Fig. 7b). Therefore, the fermentation of TWF083 was conducted in medium with a second addition of glucose, using TWF001 as a control (Fig. 7). TWF001 and TWF083 cells were grown in medium with 30 g/L initial glucose, and 30 g/L glucose was added when the initial glucose was depleted at 15 and 18 h, respectively. The highest l-threonine production in TWF001 and TWF083 reached 19.67 and 23.11 g/L, respectively (Fig. 7d). TWF001 and TWF083 cells were grown in medium with 40 g/L initial glucose, and 40 g/L glucose was added when the initial glucose was depleted at 18 h. The highest l-threonine production in TWF001 and TWF083 reached 18.90 and 29.73 g/L, respectively (Fig. 7e). TWF001 and TWF083 cells were grown in medium with 50 g/L initial glucose, and 30 g/L glucose was added when the initial glucose was depleted at 21 h. The highest l-threonine production in TWF001 and TWF083 reached 18.72 and 7.33 g/L, respectively (Fig. 7f). Compared with the control TWF001, TWF083 grown better and produced more l-threonine under the same growth condition.

**Fig. 8:**
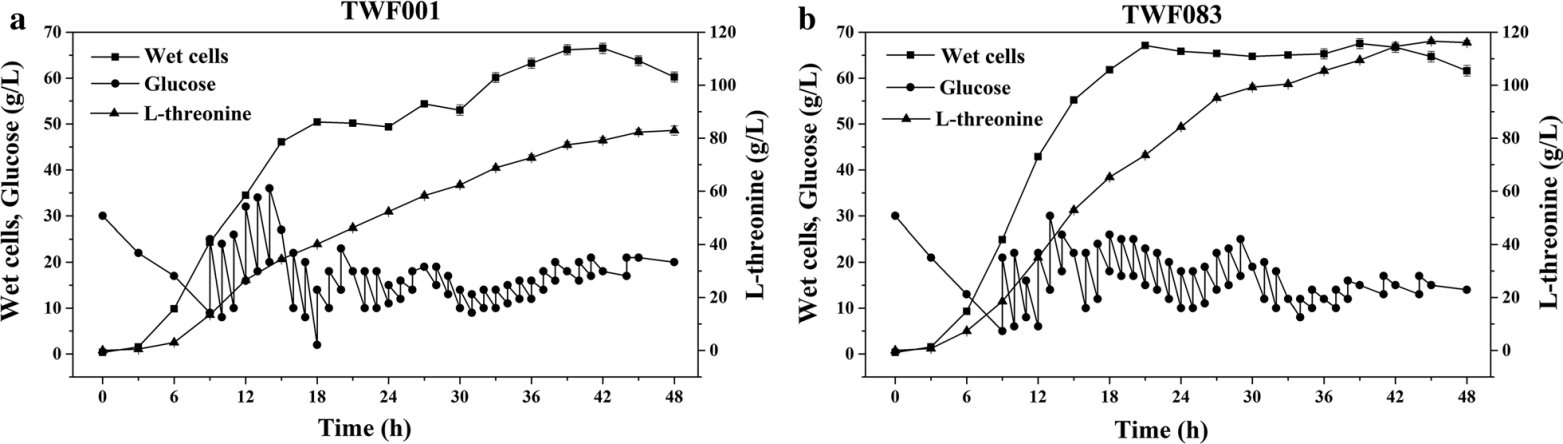
Fed-batch fermentation of TWF083, using TWF001 as a control. The error bars indicate the standard deviations from three independent experiments

Fed‐batch fermentation of TWF083 was performed in 2.4-L quadruple fermenters with a working volume of 1 L, using TWF001 as a control (Fig. 8). TWF001 could produce 82.9 g/L l‐threonine in 48 h, leading to a yield of 0.357 g/g glucose and a volumetric productivity of 1.73 g/L/h. TWF083 produced 116.62 g/L l‐threonine with a yield of 0.486 g/g glucose and productivity of 2.43 g/L/h. Both biomass accumulation and glucose consumption of TWF083 were faster than TWF001 in the fermenter, suggesting that the dynamic regulation expression of multiple key genes in TWF083 functions well in large-scale fermentation.

## Discussion

In this study, the *thrL* regulatory region was used as dynamic regulation element to control the expression of some key genes responsive to l-threonine. The gene *arcA* in TCA cycle, the genes *fadR*, *cpxR* and *gadE* in fatty acid synthesis and the gene *pykF* in aerobic respiration were repressed using the *thrR* dynamic regulation. The RBSs with different strength in *thrR* were designed to optimize the dynamic regulation of the gene *iclR* in the glyoxylate shunt. The gene *aspC* following same strength RBSs and different threonine-activating promoters, *P*_*cysH*_, *P*_*cysJ*_ and *P*_*cysD*_, were inserted into the chromosome to enhance its expression. Finally, all regulations were integrated into one strain, and the resulting strain TWF083 could produce 26.50 g l-threonine from 40 g glucose, which is 49.4% higher than the control TWF001, and the conversion rate reached 0.66 g/g (Fig. 7b). In recent years, many studies have focused on designing genetic circuits to realize dynamic regulation of metabolic flux for producing intermediates [57] such as lycopene [58], myoinositol [59] and l-lysine [60]. A series of threonine-responsive sensors were derived by transforming *thrL* leader regulatory elements, which were in the negative correlation with l-threonine concentration and has different sensitivity [61]. However, there was no report on the application of *thrL* regulation in l-threonine production.

The modification of glyoxylate shunt has been used to overcome acetate overflow and improve the production of acetyl-CoA-derived chemicals by deleting *iclR* in *E. coli* [9, 22–24, 26]. In this study, the strain TWF056, TWF057, TWF058, TWF059, TWF060, TWF061, TWF062, TWF063, TWF064 and TWF065 were constructed by inserting the different part of attenuator region of *thr* operon, *thrL*, into the different native regulatory regions in front of *iclR.* All these strains could produce more l-threonine than the control TWF001 after 30 h fermentation. TWF063 produced 11.7% more l-threonine than the *iclR* deletion mutant TWF003 [9], and its growth was also improved (Fig. 3).

The aspartate aminotransferase encoded by *aspC* draws the carbon flux from TCA cycle to the biosynthetic pathway of l-aspartate family. Deletion of *aspC* led to generation of small cells with slow growth, while overexpression of *aspC* exerted the opposite effect [55]. Using a switch to make the glycolytic fluxes towards TCA cycle in the early stage, and to make the carbon flux be redirected into l-threonine synthetic pathway by inducing the expression of *aspC*, *fdh*, *gdhA* and *pntAB* in the production stage could increase l-threonine production [62]. The gene *aspC* following different threonine-enhancing promoters were inserted into the chromosome of TWF063, resulting in the strains TWF066, TWF067 and TWF068. Because of the high expression of *aspC* in early stage, the growth of TWF067 and TWF068 was inhibited and less l-threonine was produced, but the lower expression of *aspC* at the early stage and the higher expression at the later stage made the growth of TWF066 unaffected, but the l-threonine production increased by 7% (Fig. 4).

The global regulation factor *arcA* deletion has been used in *E. coli* to activate the TCA cycle and aerobic respiration [46, 63]. The global regulation factor *fadR* deletion has been used to increase the acetyl-CoA supply by enhancing the fatty acid degradation [6]. The role of *cpxR* and *gadE* in fatty acid synthesis is to activate *fabA* and *fabZ* [32, 33], which are not regulated by *fadR*, but there was no report on the accumulation of substances by knocking out *cpxR* and *gadE.* In *pykF* knockout mutant, less acetate and more l-threonine was produced [47, 48]. With the dynamic regulation, the nature regulatory regions of the above 5 genes were replaced by *thrL* leader regulatory elements in TWF001, resulting in the strains TWF051, TWF052, TWF053, TWF054 and TWF055. All these mutants could produce more l-threonine than the control TWF001 after 30 h fermentation. TWF066 was used as a starting strain, step by step, to carry out multiple knockout test on the 5 genes to find the optimal strain. The results showed that the genetic engineering through the dynamic regulation had little effect on the metabolism of the strain in the early stage. After several rounds of modifications, the growth of strain TWF083 was similar to that of original strain TWF001 but the l-threonine production increased by 49.4%.

Several l-threonine producing *E. coli* strains have been constructed. An l-methionine auxotroph strain KY10935 obtained by a classical selection method without the use of directed genetic-engineering modifications could produce 100 g/L l-threonine after 77 h cultivation [65]. TH28C/pBRThrABCR3 constructed from W3110 by systems metabolic engineering and in silico flux response analysis could produce threonine with a high yield of 0.393 g/g glucose and 82.4 g/L threonine by fed-batch culture [8]. KCCM 10353 could produce 112 g/L l-threonine with a high yield of 0.452 g/g glucose after 77 h cultivation [66]. EC125 could produce 105.3 g/L l-threonine with a yield of 0.405 g/g glucose after 48 h two-stage feeding cultivation [67]. THRDΔ*pykF* produced less acetate (52%) and produced 112.57 g/L l-threonine with a yield of 0.376 g/g glucose after 40 h cultivation [48]. TRFC derived by repeated compound mutagenesis (DES plus UV) from *E. coli* K12 could use sucrose as the carbon source, it could produce 124.57 g/L l-threonine after 40 h cultivation, using the combined feeding strategy of pseudo-exponential feeding and glucose-stat feeding resulted in high cell density [68]. THPE5 uses a switch to make the glycolytic fluxes towards TCA cycle in the early stage, leading to the improved glucose utilization and growth performance; in the production stage the carbon flux is redirected into l-threonine synthetic pathway via a synthetic genetic circuit by inducing the expression of *aspC*, *fdh*, *gdhA* and *pntAB* to increase l-threonine production, resulting in 70.5 g/L l-threonine with a yield of 0.404 g/g glucose after 40 h cultivation [62]. JLTHR could produce 127.3 g/L l-threonine with a glucose conversion rate of 58.12% after adding betaine hydrochloride in the medium [4]. There are a few l-threonine producing strains were developed from the same origin to TWF083. TWF044 could enhance glyoxylate shunt pathway and produce 103.89 g/L l-threonine with a yield of 0.450 g/g glucose after 48 h fed-batch fermentation [6]. TWF006/pFW01-thrA*BC-*asd* could produce only 15.85 g/L l-threonine but showed a high yield of 0.528 g/g glucose after 36 h shake flask cultivation [9]. TWF018 could produce 25.1 g/L l-threonine with a high yield of 0.628 g/g glucose after 36 h shake flask cultivation [46]. WMZ016/pFW01-thrA*BC-*rhtC* derived from MG1655 by modifying the glucose specific phosphotransferase system could produce 17.98 g/L l-threonine with a yield of 0.346 g/g glucose after 36 h shake flask cultivation [69]. When TWF083 was cultured in shake flask, it produced 16.50 g/L l-threonine with a yield of 0.662 g/g glucose after 30 h, which is better than other strains derived from the same origin. After 48 h fed-batch fermentation, TWF083 could produce 116.62 g/L l-threonine with a yield of 0.486 g/g glucose.

NADPH is essential for the catalytic activity of glutamate dehydrogenase in *E. coli* [70]. It also plays a key role in l‐threonine biosynthesis due to the NADPH‐dependent β‐aspartate semialdehyde dehydrogenase and homoserine dehydrogenase [71, 72]. In *E. coli*, NADPH is mostly produced in the pentose phosphate pathway by glucose-6-phosphate dehydrogenase encoded by the gene *zwf*, which catalyzes the oxidation of glucose 6-phosphate to 6-phosphoglucono-δ-lactone; 6-phosphogluconate dehydrogenase encoded by the genes *gnd* could also produce NADPH through the oxidative decarboxylation of 6-phosphogluconate to ribulose 5-phosphate [73]. Several approaches to increase the NADPH yield through metabolic engineering of genes involved in the pentose phosphate pathway have been reported [74–80]. Therefore, it is worth studying the influence of NADPH on l-threonine production in TWF083.

In this study, a range of approaches have been proposed for the construction of l-threonine producing strain using dynamic regulation. The successful application of threonine-responsive elements to improve l-threonine production in this work provides further opportunities for synthetic biology and metabolic engineering in *E. coli*.

## Data Availability

All data generated or analyzed during this study are included in the manuscript.
